# Hepatitis C virus infection restricts human LINE-1 retrotransposition in hepatoma cells

**DOI:** 10.1371/journal.ppat.1009496

**Published:** 2021-04-19

**Authors:** Anja Schöbel, Van Nguyen-Dinh, Gerald G. Schumann, Eva Herker

**Affiliations:** 1 Institute of Virology, Philipps-University Marburg, Marburg, Germany; 2 Division of Medical Biotechnology, Paul-Ehrlich-Institute, Langen, Germany; University of California, San Diego, UNITED STATES

## Abstract

LINE-1 (L1) retrotransposons are autonomous transposable elements that can affect gene expression and genome integrity. Potential consequences of exogenous viral infections for L1 activity have not been studied to date. Here, we report that hepatitis C virus (HCV) infection causes a significant increase of endogenous L1-encoded ORF1 protein (L1ORF1p) levels and translocation of L1ORF1p to HCV assembly sites at lipid droplets. HCV replication interferes with retrotransposition of engineered L1 reporter elements, which correlates with HCV RNA-induced formation of stress granules and can be partially rescued by knockdown of the stress granule protein G3BP1. Upon HCV infection, L1ORF1p localizes to stress granules, associates with HCV core in an RNA-dependent manner and translocates to lipid droplets. While HCV infection has a negative effect on L1 mobilization, L1ORF1p neither restricts nor promotes HCV infection. In summary, our data demonstrate that HCV infection causes an increase of endogenous L1 protein levels and that the observed restriction of retrotransposition of engineered L1 reporter elements is caused by sequestration of L1ORF1p in HCV-induced stress granules.

## Author summary

Members of the Long Interspersed Nuclear Element 1 (LINE-1, L1) class of retrotransposons account for ~17% of the human genome and include ~100–150 intact L1 loci that are still functional. L1 mobilization is known to affect genomic integrity, thereby leading to disease-causing mutations, but little is known about the impact of exogenous viral infections on L1 and *vice versa*. While L1 retrotransposition is controlled by various mechanisms including CpG methylation, hypomethylation of L1 has been observed in hepatocellular carcinoma tissues of hepatitis C virus (HCV)-infected patients. Here, we demonstrate molecular interactions between HCV and L1 elements. HCV infection stably increases cellular levels of the L1-encoded ORF1 protein (L1ORF1p). HCV core and L1ORF1p interact in ribonucleoprotein complexes that traffic to lipid droplets. Despite its redistribution to HCV assembly sites, L1ORF1p is dispensable for HCV infection. In contrast, retrotransposition of engineered L1 reporter elements is restricted by HCV, correlating with an increased formation of L1ORF1p-containing cytoplasmic stress granules. Thus, our data provide first insights into the molecular interplay of endogenous transposable elements and exogenous viruses that might contribute to disease progression *in vivo*.

## Introduction

Among all endogenous transposable elements present in the human genome, the non-LTR retrotransposon LINE-1 (Long Interspersed Nuclear Element-1, L1) is the only autonomous retroelement that is currently mobilized. In total, ~17% of the human genome are comprised of L1 sequences [1]. While the majority of L1 loci are not functional due to 5’ truncations, internal rearrangements, and point mutations, approximately 100–150 L1 loci in the human genome are still functional, thus retrotransposition-competent [2–4]. A functional human L1 element is ~6 kb in length, harbors 5’ and 3’ untranslated regions (UTRs), two open reading frames (ORF1, ORF2) that are separated by a 63-bp spacer region, a poly A-tail at its 3’ end, and is flanked by variable-length target site duplications (TSDs) [5]. Additionally, a primate-specific antisense ORF0 of unknown function has been identified recently in the 5’ UTR [6]. L1ORF1p is a 40 kDa RNA-binding protein with chaperone activities [7–9] and the 150 kDa L1ORF2p harbors endonuclease (EN) as well as reverse transcriptase (RT) activities [10–12]. Both proteins are required for L1 retrotransposition and assemble with their encoding L1 mRNA in *cis*, forming a cytoplasmic ribonucleoprotein particle (L1 RNP), that represents a retrotransposition intermediate [7,13–18]. L1 RNPs associate with cytoplasmic stress granules and processing bodies (P-bodies) [18–21], but the role of these cellular structures in the L1 life cycle is still controversial. L1 RNPs, but also L1ORF1p alone, colocalize with various other RNA-binding proteins and for many of these proteins an RNA-dependent interaction with L1ORF1p has been reported [18,19,21–26]. The retrotransposition cycle also involves trafficking of L1 RNPs into the nucleus, which requires the endosomal sorting complex required for transport (ESCRT) [27]. Once inside the nucleus, the EN domain of L1ORF2p nicks the genomic DNA at the consensus target sequence 5’-TTTT/AA-3’ [11,28,29]. The resulting free 3’ OH is used by the RT domain of L1ORF2p to initiate reverse transcription of the L1 mRNA, followed by re-integration of the generated cDNA into the genome. This combined process is termed “target-primed reverse transcription” (TPRT) [30,31]. Although L1ORF2p displays a strong *cis* preference for its encoding L1 transcript, non-autonomous retroelements, such as the short interspersed nuclear elements (SINEs) *Alu* and SVA (SINE/VNTR/Alu), but also cellular mRNAs are mobilized by the L1-encoded protein machinery in *trans* occasionally [13–15,32,33]. As L1-mediated retrotransposition events can affect genome integrity and host gene expression, and lead to disease-causing mutations [34], L1 activity is suppressed in most somatic tissues by different cellular mechanisms, including antiviral host proteins [35] and epigenetic mechanisms such as CpG methylation [36–39]. Hypomethylation of CpG islands in the L1 5’ UTRs of cancerous tissues, including hepatocellular carcinoma (HCC), has been reported, and correlates with the frequency of endogenous L1 retrotransposition events [40–44]. Tumor-specific L1 retrotransposition has recently been observed in HCC and L1-mediated mobilization has been identified as an important etiological factor in HCC [40]. The predominant cause for HCC development is chronic hepatitis B virus (HBV) or hepatitis C virus (HCV) infection [45]. In this context, a recently published study reported a strong decrease in L1 methylation in HCV-related cirrhosis and HCC compared to the respective alcoholic-induced malignancies and normal liver tissue [44], suggesting a connection between HCV infection and L1 methylation.

Currently, 71 million people worldwide are estimated to be viraemic for HCV and about 400 000 patients die annually due to HCV-related hepatitis and chronic disease progression (Global Hepatitis Report 2017. Geneva: World Health Organization; 2017). HCV is a bloodborne enveloped single-stranded RNA virus within the *Flaviviridae* family. At the molecular level, the positive-sense viral genome harbors one ORF, encoding a single precursor polyprotein that is subsequently processed by cellular and viral proteases into the individual structural (the capsid protein core and the envelope glycoproteins E1 and E2) and non-structural (p7, NS2, NS3, NS4A, NS4B, NS5A, and NS5B) HCV proteins. The HCV RNA replicates in characteristic vesicular membrane structures, mainly double membrane vesicles (DMVs) [46,47], which likely originate from ER rearrangements induced by HCV. These replication vesicles include the viral proteins of the replicase complex (NS3–NS5B) and are located proximal to cytosolic lipid droplets in HCV-replicating and HCV-infected cells [48,49]. Lipid droplets, the major cellular storage organelles for neutral lipids, are an essential hub for HCV assembly [48]. A crucial prerequisite for efficient HCV particle production is the translocation of the capsid protein core and the multifunctional non-structural protein NS5A to lipid droplets [48,50–52]. Along with the viral proteins, HCV recruits various host proteins of different function to its replication sites in order to facilitate RNA replication and virus production in a favorable environment. In this context, proteins found in cellular RNP complexes, such as stress granules and P-bodies, are located in close proximity to lipid droplets in HCV-infected cells [53–57]. Recently, our group found profound changes in the lipid droplet proteome of HCV-infected cells through quantitative mass spectrometry analysis [53]. Strikingly, we exclusively identified L1ORF1p peptides in lipid droplet fractions of HCV-infected Huh7.5 hepatoma cells. As both HCV infection and L1 mobilization are important drivers of HCC development [40,45], we investigated the molecular interplay between HCV infection and L1 expression and retrotransposition. We found that HCV infection results in redistribution of L1ORF1p from cytoplasmic foci to lipid droplets, increases L1 mRNA and protein expression, but also interferes with retrotransposition of engineered L1 reporter elements.

## Results

### HCV infection causes a redistribution of L1ORF1p to lipid droplets

**Fig 1:**
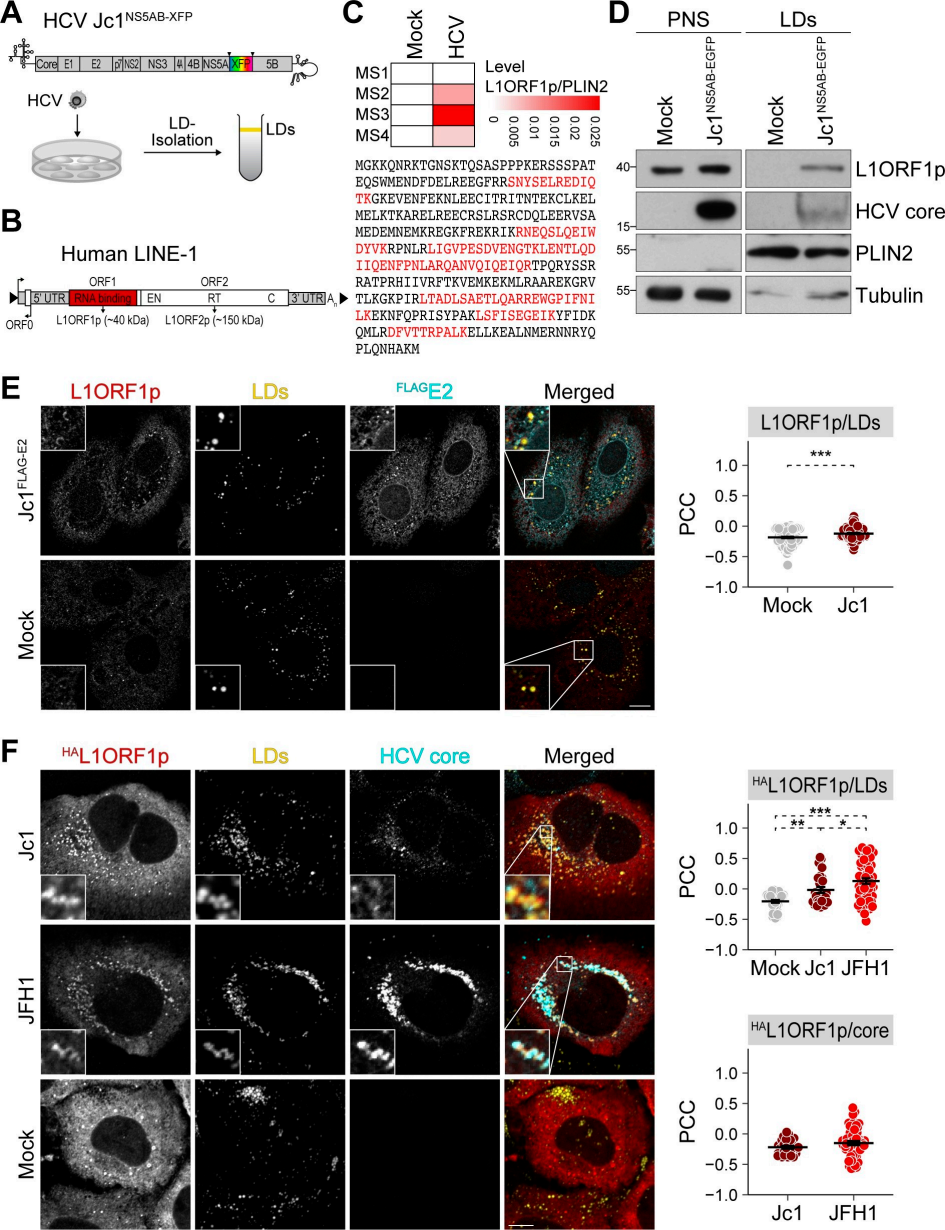
HCV infection re-localizes L1ORF1p to lipid droplets. (A) Experimental setup to identify lipid droplet–associated proteins. HCV Jc1 reporter constructs encode fluorescent proteins (XFP) flanked by duplicated NS5A-NS5B cleavage sites (S1 Fig). NS, non-structural protein. HCV-infected and uninfected cells were lysed and lipid droplets were isolated by sucrose density centrifugation. Proteins from the floating lipid droplet fraction were analyzed by mass spectrometry or immunoblotting. (B) Scheme depicts the organization of a functional endogenous L1 harboring ORF0, ORF1 (red box), and ORF2 encoding the RNA-binding protein L1ORF1p and L1ORF2p, a protein harboring endonuclease (EN) and reverse transcriptase (RT) activities, and a cysteine-rich domain (C). UTR, untranslated region; A_n_, polyA tail; arrows indicate transcriptional start sites. (C) Identification of L1ORF1p in lipid droplet fractions isolated from HCV-infected Huh7.5 cells by tandem mass spectrometry. Dataset from Rösch *et al*. [53]. Heatmap depicts L1ORF1p levels normalized to PLIN2 measured in four independent quantitative mass spectrometry (MS) experiments. L1ORF1p-specific peptides identified by mass spectrometry are highlighted in red as part of the presented L1ORF1p protein sequence. (D) Immunoblot analysis of lipid droplet fractions isolated from Jc1^NS5AB-EGFP^-infected Huh7.5 cells (multiplicity of infection (MOI) 0.02) at 9 days post infection (dpi) demonstrates localization of endogenous L1ORF1p to lipid droplets in HCV-infected cells. Tubulin and PLIN2 served as loading controls for post-nuclear supernatants (PNS) and lipid droplets (LDs), respectively. Shown is one representative experiment (n = 3). (E) Analysis of endogenous L1ORF1p localization in HCV-infected and uninfected Huh7.5 cells by confocal microscopy. HCV Jc1^FLAG-E2^ and mock-electroporated cells were fixed at 3 days post electroporation (dpe) and stained using L1ORF1p and FLAG antibodies. Lipid droplets (LDs) were visualized with BODIPY655/676. Shown are representative images (scale bar 10 μm). Colocalization of endogenous L1ORF1p with lipid droplets was analyzed by calculating the Pearson’s correlation coefficients (PCC) of individual cells from 2 independent experiments (# of cells: mock = 119, Jc1^FLAG-E2^ = 109; mean ± SEM, ***p< 0.001, Welch’s *t*-test). (F) HCV infection causes partial colocalization of ^HA^L1ORF1p with HCV core at lipid droplets. HCV Jc1 (MOI 0.03), JFH1 (MOI 0.02), and mock-infected Huh7 cells were transfected with the ^HA^L1ORF1p expression plasmid at 7 dpi. 2 days post transfection (dpt), cells were fixed and stained using HCV core and HA antibodies; lipid droplets were visualized with BODIPY493/503. Shown are representative images (scale bar 10 μm). Colocalization analysis of ^HA^L1ORF1p with lipid droplets or HCV core was performed by calculating the PCC of individual cells from 2 independent experiments (# of cells: mock = 42, Jc1 = 26, JFH1 = 52; mean ± SEM, *p< 0.05, **p< 0.01, ***p< 0.001, Welch’s *t*-test).

Lipid droplets are of major importance for HCV infection, serving as putative assembly sites for progeny virions, and host factors are recruited to lipid droplets upon infection providing a favorable environment for viral replication. In a recently reported quantitative lipid droplet proteome analysis of HCV-infected and uninfected hepatoma cells, we found a number of host proteins recruited to lipid droplets upon infection [53]. Interestingly, we identified peptides of the L1-encoded ORF1 protein (L1ORF1p) in purified lipid droplet fractions from HCV-infected Huh7.5 cells in three out of four independent experiments, but we never detected them in lipid droplet fractions from uninfected control cells (Fig 1A–1C).

In order to confirm the localization of L1ORF1p to lipid droplet fractions, we infected hepatoma cells with an HCV Jc1 EGFP reporter virus (Jc1^NS5AB-EGFP^, viral constructs are listed in S1 Fig) [58] and isolated lipid droplets by sucrose gradient centrifugation followed by immunoblot analysis. Consistent with the results of the mass spectrometry analysis [53], we detected L1ORF1p in lipid droplet fractions from HCV-infected cells, but not from mock-infected controls, although they also expressed endogenous L1ORF1p (Fig 1D). Next, we visualized and compared endogenous L1ORF1p localization in HCV-infected and mock-infected Huh7.5 cells using confocal microscopy (Fig 1E). In mock-infected cells, L1ORF1p displayed a predominantly dotted cytoplasmic localization (Fig 1E). In cells infected with an HCV Jc1^FLAG-E2^ strain [59], we observed a partial redistribution of L1ORF1p to half-ring-shaped patterns surrounding individual lipid droplets (Fig 1E). We then quantified colocalization of L1ORF1p with lipid droplets by calculating the Pearson’s correlation coefficient (PCC) (Fig 1E) as well as the Manders’ colocalization coefficient (MCC) (S2A Fig). The MCCs M1 and M2 can be defined as the fraction of channel 1 that overlaps with channel 2 and *vice versa*. In contrast, PCC calculates the overall correlation of signal intensities between two channels. HCV infection slightly increased the colocalization of L1ORF1p and lipid droplets compared to mock-infected cells (Fig 1E). However, the MCC analysis was inconclusive due to the low signal intensity of endogenous L1ORF1p staining (S2A Fig). Therefore, we overexpressed an HA-tagged L1ORF1p (^HA^L1ORF1p) (S1 Fig) in HCV-infected cells and additionally performed colocalization analysis of lipid droplet-localized HCV core with ^HA^L1ORF1p. As observed for endogenous L1ORF1p, ^HA^L1ORF1p predominantly localized to cytoplasmic foci in mock-infected cells (Fig 1F), which is consistent with the previously reported subcellular localization of L1ORF1p overexpressed from transiently transfected L1 reporter elements [18,19,23]. For infection of Huh7 cells, we chose two different HCV strains: the chimeric cell culture–adapted strain Jc1 [60], and JFH1, a molecular clone of an HCV isolate [61,62]. JFH1 spreads less efficiently between cells compared to Jc1, but infected cells display more lipid droplet–localized HCV capsid protein core in the perinuclear region [63]. ^HA^L1ORF1p localized to lipid droplets in both Jc1 and JFH1-infected cells (Fig 1F). While ^HA^L1ORF1p displayed a more punctate staining in lipid droplet–rich areas in Jc1-infected cells, we observed a more defined, even half-ring-shaped localization of ^HA^L1ORF1p surrounding lipid droplets in JFH1-infected cells that correlated with the core protein levels located at lipid droplets (Fig 1F). PCC and MCC analyses indicate that colocalization of ^HA^L1ORF1p with lipid droplets was slightly increased in Jc1-infected cells and more pronounced in JFH1-infected cells compared to uninfected controls (Figs 1F and S2B). However, we observed only a partial colocalization of HCV core and ^HA^L1ORF1p, with a mean M1 (^HA^L1ORF1p) of approximately 0.2 for Jc1 and JFH1, and M2 (core) of 0.3 for Jc1 and 0.4 for JFH1.

### HCV replication increases endogenous L1ORF1p levels

**Fig 2:**
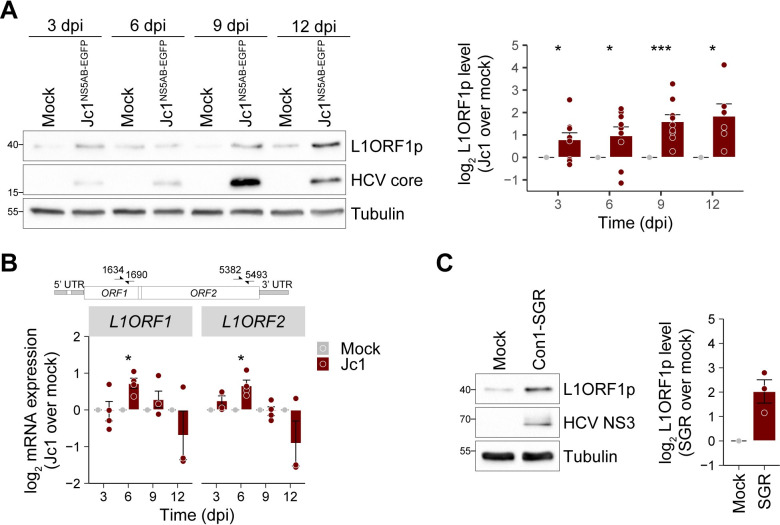
HCV infection increases L1ORF1p protein levels. (A) L1ORF1p protein levels in mock and Jc1^NS5AB-EGFP^-infected Huh7.5 cells (MOI 0.2) were analyzed by immunoblotting at 3–12 dpi and quantified by densitometry. Shown is the relative L1ORF1p level normalized to tubulin expression (mean ± SEM, n = 6–9, *p< 0.05, ***p< 0.001, Welch’s t-test) that demonstrates a strong increase of L1ORF1p expression in HCV-infected cells. (B) HCV infection transiently increases expression of endogenous full-length L1 elements. Time course of L1 expression after infection of Huh7.5 cells with Jc1^NS5AB-EGFP^ (MOI 0.2). Endogenous L1 mRNA levels were determined by qRT-PCR using RNA isolated at the indicated time points and primer pairs binding within ORF1 or ORF2 (mean ± SEM, n = 3–4, *p< 0.05, Welch’s *t*-test). Primer binding sites within L1 sequences are marked in the scheme. (C) L1ORF1p levels in stable polyclonal Huh7.5-Con1-SGR cells were analyzed by immunoblotting and quantified. Shown is the relative L1ORF1p level normalized to tubulin (mean ± SEM, n = 3).

In the initial experiments, we noted a slight increase of endogenous L1ORF1p in post-nuclear supernatants of HCV-infected Huh7.5 cells (Fig 1D). Following this observation, we performed time-course experiments to assess endogenous L1ORF1p expression upon infection with HCV. L1ORF1p levels steadily increased after infection reaching a ~4-fold accumulation of L1ORF1p in HCV-infected relative to mock-infected cells at 12 dpi (Fig 2A). Using primers that bind in the ORF1 and ORF2 region of L1, we quantified endogenous full-length L1 mRNA expression levels and observed only a transient ~2-fold increase of L1 mRNA levels relative to mock-infected cells that declined again after 6 dpi (Fig 2B). An explanation for these results could be that the L1ORF1p immunoblot analysis measures expression from intact genomic L1 loci encoding functional L1ORF1p, while the qRT-PCR analysis detects L1 transcripts expressed from both intact and mutated L1 loci, which do not express functional L1ORF1p. Expression of intact genomic L1 loci is regulated by methylation of CpG islands located in the L1 5’ UTRs and CpG hypomethylation was previously detected in HCV-related cirrhosis and HCC [44]. Furthermore, HCV infection has been described to induce persistent epigenetic changes that are associated with an increased HCC risk in patients [64]. However, in our infection system using Huh7-derived cells, analysis of the methylation state of L1 5’ UTR regions did reveal only partial methylation in Huh7.5 cells without any changes after HCV infection (S3 Fig). These findings suggest that the elevated L1ORF1p levels in our system do not result from an overall upregulation of transcription of full-length L1 elements. L1ORF1p levels were also increased by ~2 fold in stable Con1 subgenomic replicon (Con1-SGR) [65] Huh7.5 cells, expressing a bicistronic RNA that lacks the structural HCV proteins as well as p7 and NS2 and encodes a neomycin resistance gene for selection (Huh7.5-Con1-SGR cells) (Fig 2C). This indicates that active HCV RNA replication or expression of the non-structural HCV proteins account for increased L1ORF1p levels.

### HCV infection restricts L1 retrotransposition

**Fig 3:**
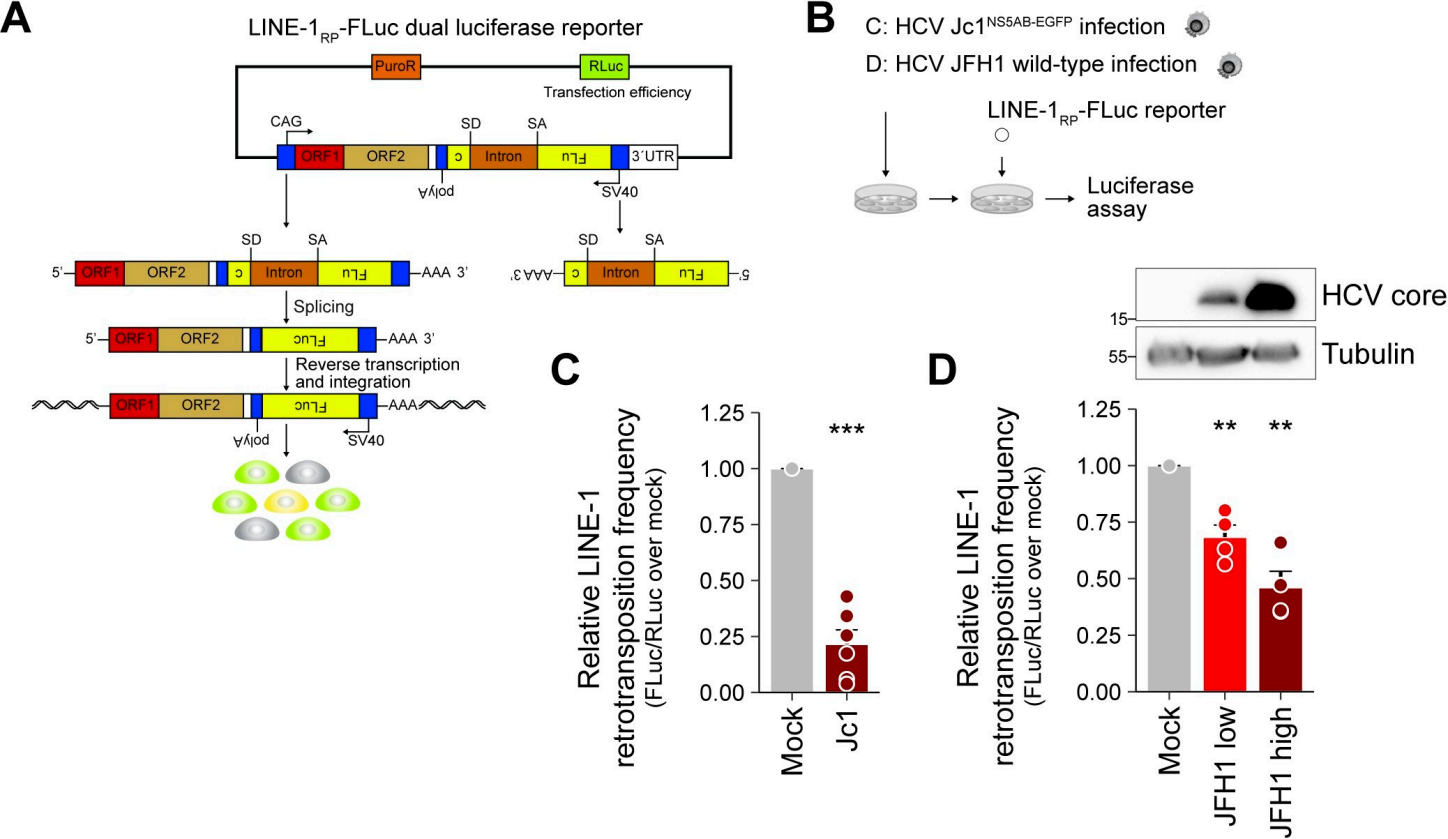
HCV infection decreases L1 retrotransposition frequency. (A) Scheme of the dual-luciferase L1 retrotransposition reporter assay expressing *Renilla* luciferase (RLuc) for transfection normalization and firefly luciferase (FLuc) as reporter for L1 retrotransposition (L1_RP_-FLuc). The FLuc gene is in antisense orientation and interrupted by an intron in sense orientation flanked by splice donor (SD) and acceptor (SA) sites ensuring that FLuc is expressed only after splicing of its pre-mRNA, reverse transcription, and integration of an intact Fluc cDNA copy. Thus, FLuc activity is directly proportional to the number of L1_RP_-FLuc *de novo* retrotransposition events. (B) Scheme of the experiments; the indicated cells were transfected with the L1_RP_-FLuc reporter and luciferase assays were performed. (C) Mock or Jc1^NS5AB-EGFP^-infected Huh7.5 cells (MOI 0.2) were transfected with L1_RP_-FLuc at 7 dpi. Depicted is the relative L1 retrotransposition frequency 5 days after duplicate transfections from 3 independent experiments (mean ± SEM, n = 6, ***p< 0.001, Welch’s *t*-test). (D) JFH1-infected Huh7 cells were cultured for ~6 months. Mock and JFH1-infected cells with low and high infection rates were transfected with L1_RP_-FLuc. Shown is the relative L1 retrotransposition frequency 6 dpt of duplicate transfections from 2 independent experiments (mean ± SEM, n = 4, **p< 0.01, Welch’s *t*-test). Immunoblot analysis of HCV core expression confirmed differences in infection rates.

Although L1 activity during early development and its role in human disease were investigated in numerous studies, little is known about L1 mobilization in the context of viral infections. Using an L1 retrotransposition reporter assay that is based on dual-luciferase activity [66] (Fig 3A), we compared L1 retrotransposition rates in mock-infected and HCV-infected Huh7.5 cells (Fig 3B). Following infection with Jc1^NS5AB-EGFP^, we transfected the cells with the L1 retrotransposition reporter plasmid L1_RP_-FLuc (pYX017) [66] and quantified L1 retrotransposition activity at 5 days post transfection. Strikingly, we observed a significant decrease in marked L1 retrotransposition frequency in HCV Jc1-infected cells by ~75% relative to mock-infected cells (Fig 3C). To assess whether the L1 retrotransposition frequencies obtained from the engineered L1-FLuc reporter assay correlate with the number of marked genomic L1-FLuc *de novo* insertions, we performed a second set of L1 reporter assays, and this time isolated genomic DNA to quantify the number of L1-FLuc *de novo* retrotransposition events by qRT-PCR (S4 Fig). Consistent with the reduction of L1 retrotransposition frequency measured by luciferase activity, the number of genomic L1-FLuc *de novo* insertions were reduced by ~75% in Jc1-infected cells compared to mock-infected cells (S4 Fig), confirming that the reduced firefly luciferase signal in HCV-infected cells reflects a reduced number of L1-FLuc retrotransposition events.

We additionally validated our results using an alternative L1 retrotransposition reporter assay, in which EGFP expression instead of firefly luciferase activity served as reporter for L1 retrotransposition [67] (S5 Fig). To this end, we transfected Huh7.5 cells 2 and 7 days post HCV Jc1 infection, and again observed a marked decrease in L1 retrotransposition events in HCV-infected cells by ~75% compared to mock infected cells at late time points after infection (S5 Fig). While HCV strain Jc1 is a partially cell-culture adapted J6/JFH1 chimera that spreads rapidly, the original isolate JFH1 displays much slower infection kinetics. To model the impact of chronic HCV infection on L1 retrotransposition frequency, we analyzed L1 retrotransposition of marked L1 elements in long-term JFH1-infected Huh7 cells (Fig 3B and 3D). HCV-infected cells were transfected with the L1 dual-luciferase reporter plasmid at ~6 months post electroporation with JFH1 RNA. Interestingly, we observed an HCV infection level–dependent decrease in L1 retrotransposition with ~30% decrease in Huh7 cells with low level JFH1 infection, and a ~50% decrease in Huh7 cells with a high level JFH1 infection as indicated in the corresponding immunoblots of the cell lysates (Fig 3D).

### HCV infection does not enhance expression of L1 restricting innate immune response genes

As multiple host-encoded proteins involved in the innate antiviral response negatively regulate L1 retrotransposition [35,68], we investigated if HCV replication induces the expression of L1 restriction factors, including interferon-stimulated genes (ISGs) such as MOV10, or members of the APOBEC3 protein family [24,68–71]. However, HCV infection of Huh7 and Huh7-derived hepatoma cell lines only marginally induces ISGs [72,73]. Accordingly, we did not observe the induction of APOBEC3 protein family members in HCV-infected or Huh7.5-Con1-SGR cells (S6 Fig). Further, expression of the known L1 restriction factors ADAR1 [74], MOV10 [69,75,76], and TRIM5α [77] was decreased in HCV-infected cells and unchanged in Huh7.5-Con1-SGR cells (S6 Fig), and HCV infection did not change MOV10 protein levels (Fig 5B), indicating that L1 mobilization is not restricted by ISG induction in HCV-infected cells.

### Lipid droplet localization of L1ORF1p is linked to HCV core trafficking

**Fig 4:**
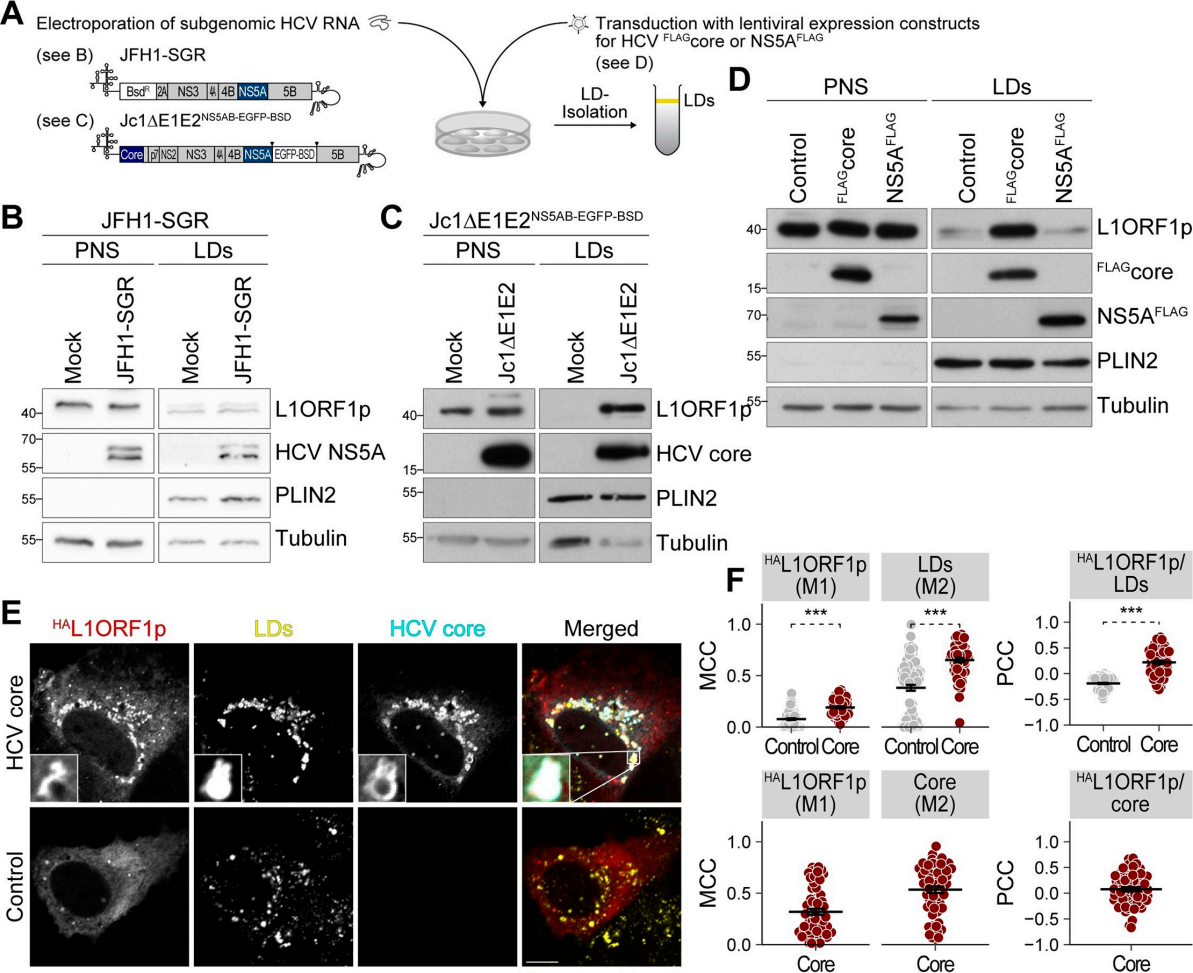
HCV core mediates L1ORF1p redistribution from cytoplasmic foci to lipid droplets. (A) Scheme of the experimental setup. Huh7.5 cells were either electroporated with JFH1 subgenomic replicon (JFH1-SGR^BSD^, gt 2a) RNA encoding NS3–NS5B (see B) or with a partially envelope-deleted Jc1 replicon Jc1ΔE1E2^NS5AB-EGFP-BSD^ (gt 2a, see C), or transduced with lentiviral expression constructs for HCV ^FLAG^core or NS5A^FLAG^ (gt 2a, see D), and lipid droplets were isolated by sucrose density centrifugation. (B–D) Immunoblot analysis of lipid droplet fractions isolated from Huh7.5 cells electroporated with JFH1-SGR^BSD^ (n = 3) (B), Jc1ΔE1E2^NS5AB-EGFP-BSD^ (n = 1) (C), or transduced with lentiviral expression constructs for ^FLAG^core or NS5A^FLAG^ (n_FLAGcore_ = 4, n_NS5AFLAG_ = 2) (D). Shown are representative experiments. Tubulin and PLIN2 served as loading controls for post-nuclear supernatants (PNS) and lipid droplets (LDs), respectively. (E) Confocal microscopy of Huh7 cells transfected with ^HA^L1ORF1p and ^FLAG^core (gt 2a) expression plasmids and stained with anti-core and anti-HA tag antibodies and BODIPY493/503 for lipid droplets. Shown are representative images (scale bar 10 μm). (F) Quantification of colocalization of ^HA^L1ORF1p with lipid droplets and ^HA^L1ORF1p with core as presented in (E). Shown are Manders’ colocalization coefficients (MCC) M1 and M2 and Pearson’s correlation coefficients (PCC) of individual cells from 3 independent experiments (# of cells: control = 91, HCV core = 61; mean ± SEM, ***p< 0.001, Welch’s *t*-test).

As we observed the distinct re-localization of L1ORF1p to lipid droplets in HCV-infected cells (Fig 1), we addressed if this redistribution can be pinpointed to a specific step in the HCV replication cycle or to specific HCV proteins. To investigate a possible effect of active HCV RNA replication and expression of NS5A, we used Huh7.5 cells electroporated with JFH1-SGR^BSD^ RNA (Fig 4A). In contrast to HCV-infected cells, we did not observe an enrichment of L1ORF1p in lipid droplet fractions of JFH1-SGR^BSD^-transfected cells compared to control cells (Fig 4B), indicating that neither active HCV RNA replication nor the expression of the HCV non-structural proteins NS3–NS5B are involved in L1ORF1p re-localization. However, replication of an envelope-deleted HCV RNA replicon (Jc1ΔE1E2^NS5AB-EGFP-BSD^) caused a redistribution of endogenous L1ORF1p to lipid droplet fractions (Fig 4A and 4C), arguing that the capsid protein core or the function of p7 and NS2 play a role in this phenotype. Two of the HCV proteins, core and NS5A, are known to strongly localize to lipid droplets in HCV-infected cells as well as upon single protein expression [48,52]; therefore, we expressed FLAG-tagged core or NS5A in Huh7.5 cells by lentiviral transduction (Fig 4A). As expected, we detected only negligible amounts of endogenous L1ORF1p in isolated lipid droplet fractions of NS5A or control lentivirus-transduced cells. In contrast, L1ORF1p was strongly enriched in lipid droplet fractions from core-expressing cells (Fig 4D). To confirm these data, we co-transfected Huh7 cells with ^HA^L1ORF1p and ^FLAG^core expression plasmids and performed immunofluorescence microscopy to visualize the distribution of ^HA^L1ORF1p, lipid droplets, and HCV core. ^HA^L1ORF1p re-localized from cytoplasmic foci to core-containing lipid droplets and quantification revealed a significantly increased colocalization of ^HA^L1ORF1p with lipid droplets and a partial direct colocalization with core (Fig 4E and 4F). To exclude HCV genotype-specific effects, we performed the same experiments with genotype 1b (gt 1b) constructs and found again that L1ORF1p was not enriched in lipid droplet fractions isolated from Con1-SGR-electroporated cells, whereas HCV core (gt 1b) expression caused a redistribution of L1ORF1p to lipid droplets (S7A–S7C Fig).

One pre-requisite of HCV core trafficking to lipid droplets is its C-terminal processing by the cellular signal peptide peptidase [78]. To determine if L1ORF1p and HCV core concomitantly traffic to lipid droplets, we took advantage of a core mutant with an unprocessable signal peptide (SPMT) [78] that is retained at the ER (S8A Fig). After subcellular fractionation, we mainly detected HCV core^SPMT^ in microsomal membrane (MM) fractions, where also the majority of L1ORF1p accumulated (S8B Fig). In contrast, core^WT^ expression led to an enrichment of both core^WT^ and endogenous L1ORF1p at lipid droplets. We confirmed these findings by immunofluorescence microscopy (S8C Fig) and observed a decreased localization of ^HA^L1ORF1p to lipid droplets in cells expressing the core SPMT mutant compared to cells expressing wild-type core (S8D Fig). Taken together, our results indicate that HCV core mediates redistribution of L1ORF1p from cytoplasmic foci to lipid droplets and that this effect is independent of viral RNA replication and expression of NS5A.

### HCV core associates with an L1ORF1p-containing ribonucleoprotein complex

**Fig 5:**
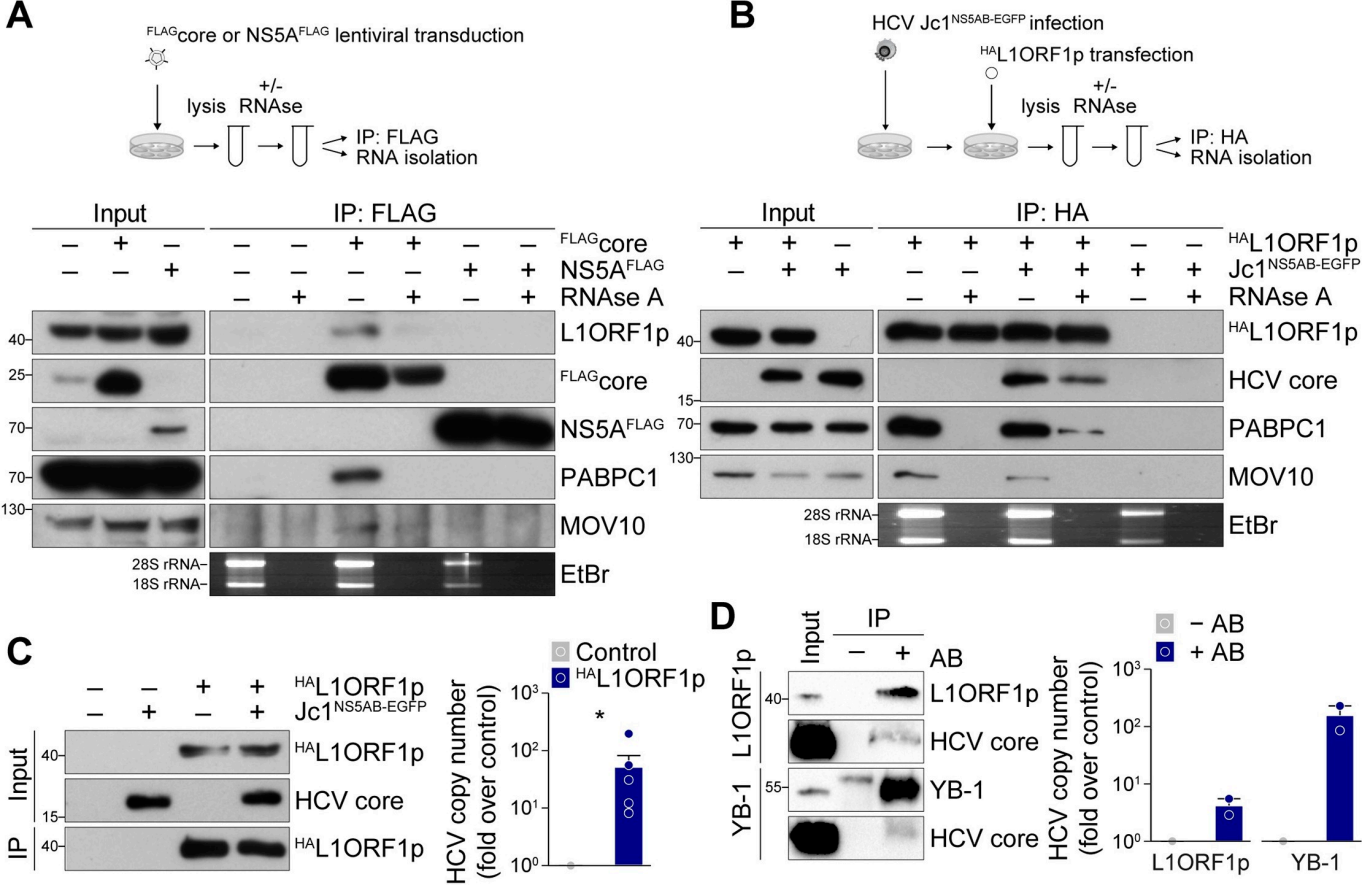
L1ORF1p interacts with HCV core in an RNA-dependent manner. (A) Co-immunoprecipitation analysis of ^FLAG^core or NS5A^FLAG^ with endogenous L1ORF1p and its interaction partners MOV10 and PABPC1. Lysates of cells transduced with lentiviral ^FLAG^core or NS5A^FLAG^ expression constructs were incubated either with RNaseOUT or RNase A followed by FLAG-specific immunoprecipitation and immunoblotting or total RNA isolation and subsequent agarose gel electrophoresis and ethidium bromide (EtBr) staining (bottom panel). Successful RNase A treatment of cell lysates was demonstrated by the absence of 18S and 28S rRNA. Shown is one representative experiment (n = 3). (B) Co-immunoprecipitation analysis of ^HA^L1ORF1p in HCV Jc1^NS5AB-EGFP^-infected or mock-infected Huh7.5 cells. Jc1^NS5AB-EGFP^-infected (MOI 0.2) or mock-infected Huh7.5 cells were transfected 7 dpi with the ^HA^L1ORF1p expression construct and lysed 2 dpt. RNAse treatment, total RNA isolation, and immunoprecipitation with HA-specific agarose beads were performed as described in (A). One representative experiment is presented (n = 3). (C) HCV RNA co-precipitates with ^HA^L1ORF1p. Relative quantification of HCV RNA copies in immunoprecipitates obtained from Jc1^NS5AB-EGFP^-infected Huh7.5 cells transiently expressing ^HA^L1ORF1p. Cells were lysed 2 dpt and 9 dpi and HA-specific immunoprecipitation was performed. Untransfected cells served as control and successful infection and expression of ^HA^L1ORF1p and HCV core as well as immunoprecipitation were confirmed by immunoblotting. HCV copy numbers were quantified by qRT-PCR using a serial dilution of *in vitro* transcribed HCV RNA as a standard (mean ± SEM, n = 6, *p< 0.05, Mann-Whitney *U* test). (D) Comparison of HCV RNA copies and interaction with HCV core in immunoprecipitates of endogenous L1ORF1p and YB-1 obtained from from Jc1^NS5AB-EGFP^-infected Huh7.5 cells. Expression and immunoprecipitation of L1ORF1p and YB-1 as well as HCV core were analyzed by immunoblotting. Immunoprecipitation without specific antibodies (-AB) served as control. HCV RNA copy numbers were determined as described in (C). Shown are relative HCV RNA copy numbers as fold over control (n = 2).

As our results suggested an HCV replication-independent effect of the HCV core protein on subcellular L1ORF1p localization, we hypothesized that both proteins directly interact with each other. To test this hypothesis, we performed co-immunoprecipitation experiments from Huh7.5 cells stably expressing ^FLAG^core or NS5A^FLAG^ (gt 2a). As L1ORF1p was reported to interact with many host-encoded proteins in an RNA-dependent manner [22–24,26] and both core and NS5A can bind RNA [79–81], we also investigated whether a putative interaction with endogenous L1ORF1p is RNA-dependent. Our data show that L1ORF1p co-purified with ^FLAG^core but not with NS5A^FLAG^ (Fig 5A). RNAse A treatment of the cell lysate prior to immunoprecipitation resulted in a decreased L1ORF1p signal (Fig 5A), indicating an RNA-dependent interaction of HCV core with L1ORF1p. In line with these results, L1ORF1p also co-purified with ^FLAG^core (gt 1b) in an RNA-dependent manner (S7D Fig). As mentioned above, L1ORF1p is known to interact with many other RNA-binding proteins in an RNA-dependent manner and is associated with proteins found in cytoplasmic stress granules and P-bodies. Comparing our previously published lipid droplet proteome analysis [53] with a recent L1ORF1p interactome study [22], we found that some L1ORF1p-interactors were also enriched in lipid droplet fractions from HCV-infected cells (22 out of 32 overlapping proteins were enriched, and 10 were decreased in lipid droplet fractions of HCV-infected cells, S9 Fig). Hence, we additionally probed the immunoprecipitates for two jointly identified proteins: the polyadenylate-binding protein 1 (PABPC1) and the RNA helicase MOV10. Similar to L1ORF1p, both proteins were found to co-precipitate with ^FLAG^core but not with NS5A^FLAG^ (Fig 5A). To confirm our results, we ectopically expressed ^HA^L1ORF1p in HCV-infected or mock-infected Huh7.5 cells and performed the reciprocal co-immunoprecipitation by pulling down HA-tagged L1ORF1p. We detected HCV core in ^HA^L1ORF1p immunoprecipitates and to a lower extent in samples precipitated from RNAse A-treated cell lysates, substantiating the RNA-dependent interaction of HCV core and L1ORF1p (Fig 5B). Immunoblot analyses confirmed the recently reported RNA-dependent interaction of PABC1 and MOV10 with L1ORF1p [22] and showed that this was independent of HCV infection (Fig 5B). Thus, we conclude that in HCV-infected cells, core is part of cellular RNP complexes that are associated with L1ORF1p explaining the observed concerted co-precipitation of HCV core with L1ORF1p, PABPC1, and MOV10.

The localization of cellular RNP complexes and P-body or stress granule proteins to HCV replication sites in infected cells has been described before [53,55–57,82]. Indeed, a unique virus-host protein complex has been suggested that is collectively redistributed to lipid droplets in HCV JFH1-expressing cells [55]. Noteworthy, the presence of HCV core seems to be required but not sufficient for redistribution of cellular RNP complexes to lipid droplets [55,56]. Based on the concerted interaction between L1ORF1p, PABPC1, MOV10, and HCV core, we speculated that HCV core not only mediates L1ORF1p redistribution but induces the re-localization of L1ORF1p-associated RNP complexes to lipid droplets in HCV-infected cells. In line with our hypothesis, we detected PABPC1 and MOV10 in lipid droplet fractions isolated from HCV core-expressing Huh7.5 cells, but not from control lentivirus-transduced or NS5A-expressing cells (S10A Fig). To substantiate our findings, we subsequently transduced Huh7.5 cells with expression constructs for HCV core and either HA-tagged wild-type L1ORF1p (^HA^L1ORF1p^WT^) or an RNA-binding mutant (^HA^L1ORF1p^Mut^; RR_261-262_AA mutant) [8,16,17] that was reported earlier to have a severely reduced ability to localize to L1 RNPs [17,18]. Immunoblot analysis of the isolated lipid droplet fractions revealed lower amounts of ^HA^L1ORF1p^Mut^ relative to ^HA^L1ORF1p^WT^ in lipid droplet fractions (S10B Fig), confirming that L1ORF1p is translocated to lipid droplets in an RNA-dependent manner as part of a larger RNP. Yet, we did not detect L1ORF2p in our lipid droplet proteome analysis [53]. This might be due to very low expression levels of endogenous L1ORF2p compared to L1ORF1p, because even L1 expression constructs in transfected cells were reported to produce L1ORF1p at up to 1000–10000-fold higher levels than L1ORF2p and expression might be restricted to a subset of cells within a population [83–86].

The presence of RNA-binding proteins or a cellular RNP complex at HCV replication sites poses the possibility that the HCV RNA genome is part of this complex. Thus, we again performed HA-specific co-immunoprecipitation from HCV-infected cells transiently expressing ^HA^L1ORF1p and determined the HCV RNA copy number in the precipitates by quantitative RT-PCR (qRT-PCR). We detected a significant enrichment of HCV genome copies in ^HA^L1ORF1p samples, suggesting that the HCV RNA is indeed part of the L1ORF1p-containing RNP complex (Fig 5C). However, from our experimental setup, we cannot conclude if L1ORF1p directly binds the viral genome or if the binding is facilitated by any of the other proteins that are part of the co-immunoprecipitated RNP complexes. The latter is not unlikely, as other P-body or stress granule-associated proteins that were previously found to localize to HCV replication sites, such as DDX3 or YB-1, also associate with the viral RNA [56,87,88]. To compare the relative amount of HCV RNA co-precipitating with L1ORF1p in relation to other RNA-binding proteins, we performed immunoprecipitation of endogenous L1ORF1p and YB-1 from HCV-infected Huh7.5 cells and determined HCV RNA copy numbers (Fig 5D). We confirmed that HCV core co-precipitated with both endogenous proteins. Again, we detected HCV RNA in L1ORF1p precipitates but the HCV RNA copy numbers were 10-fold higher in YB-1 precipitates which can be explained by the strong *cis* preference of L1ORF1p for its own mRNA. Still, L1 proteins have been reported to associate with other cellular RNAs in *trans* [13,15,22,32,33], raising the possibility that L1ORF1p binds directly to HCV RNA.

### Presence of HCV RNA 3’ UTRs impairs L1 mobilization

**Fig 6:**
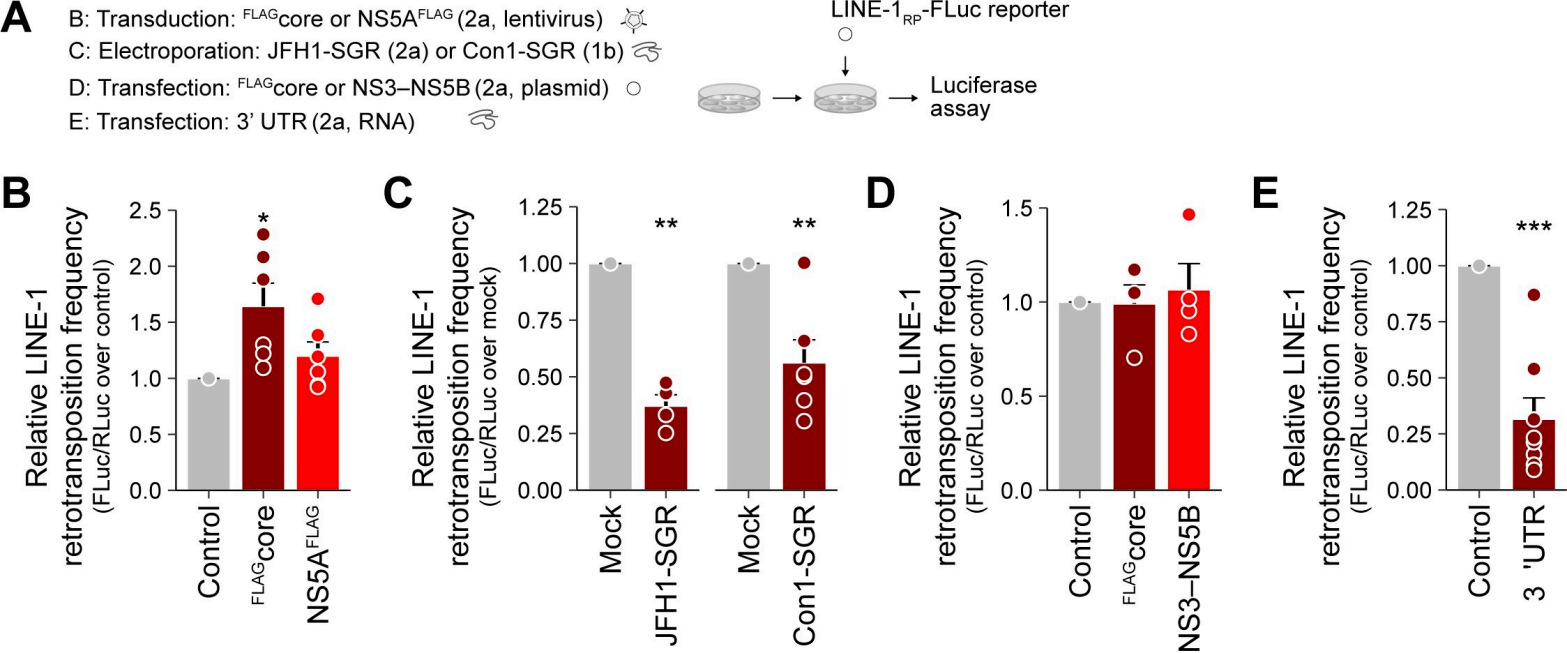
An HCV RNA-induced mechanism but not viral protein expression impairs L1 retrotransposition. (A) Scheme of the experiments; the indicated cells were transfected with the L1_RP_-FLuc reporter and luciferase assays were performed. (B) Huh7.5 cells were transduced with lentiviral expression constructs for HCV ^FLAG^core, NS5A^FLAG^, or control lentivirus-transduced. Stable expression of HCV ^FLAG^core, but not of NS5A^FLAG^ increased L1 retrotransposition frequency in Huh7.5 cells relative to control lentivirus-transduced cells. Shown is the relative L1 retrotransposition frequency at 6 dpt of duplicate transfections from 3 independent experiments (mean ± SEM, n = 6, *p< 0.05, Welch’s *t*-test). (C) L1 retrotransposition frequency in HCV SGR cells relative to naive Huh7.5 cells. Huh7.5 JFH1-SGR^BSD^ (gt 2a), Huh7.5-Con1-SGR (gt 1b) or Huh7.5 cells were transfected with L1_RP_-FLuc. Shown is the relative L1 retrotransposition frequency 5–6 dpt of duplicate transfections from 3 independent experiments (mean ± SEM, n = 6, **p< 0.01, Welch’s *t*-test). (D) Plasmid-based expression of HCV ^FLAG^core and the JFH1 NS3–NS5B (gt 2a) polyprotein does not affect L1 retrotransposition frequency in Huh7.5 cells relative to control cells. Cells were transfected with the respective plasmids 24 hours prior to transfection with the L1_RP_-FLuc reporter. Shown is the relative L1 retrotransposition frequency at 5 dpt of duplicate transfections from 2 independent experiments (mean ± SEM, n = 4). (E) Transfection with HCV 3’ UTR RNA reduces L1 retrotransposition. Huh7.5 cells were transfected with the L1_RP_-FLuc reporter plasmid and ~6 hours later, cells were additionally transfected with 0.5 μg of *in vitro* transcribed HCV 3’ UTR RNA per well. Mock-transfected cells served as control and RNA transfection was repeated 48 h later. Shown is the relative L1 retrotransposition frequency at 5 dpt of duplicate transfections from 4 independent experiments (mean ± SEM, n = 8; ***p< 0.001, Welch’s *t*-test).

Previous studies have shown that the formation of intact L1 RNPs is an important prerequisite for L1 retrotransposition [17]. Thus, we reasoned that HCV infection might disturb L1 RNP integrity or interferes with the transport of intact L1 RNPs to the nucleus, thereby causing a reduced L1 retrotransposition frequency. To test the hypothesis that HCV core–mediated re-localization of L1ORF1p to lipid droplets contributes to the reduced L1 retrotransposition we observed in HCV-infected cells, we first transduced Huh7.5 cells for overexpression of HCV core or NS5A, and subsequently transfected these cells with the L1 dual-luciferase reporter plasmid (Fig 6A and 6B). Unexpectedly, stable expression of HCV core slightly increased L1 retrotransposition frequencies relative to the NS5A and control lentivirus-transduced Huh7.5 cells (Fig 6B). Thus HCV-core mediated redistribution of L1ORF1p to lipid droplets is not sufficient to impair L1 retrotransposition in HCV-infected cells.

We then investigated if HCV RNA replication itself negatively affects L1 retrotransposition and transfected Huh7.5 cells harboring a JFH1 subgenomic replicon (JFH1-SGR^BSD^, gt 2a; S1 Fig) or Huh7.5-Con1-SGR cells with the L1 dual-luciferase reporter plasmid and performed luciferase reporter assays in presence of ongoing HCV RNA replication (Fig 6A and 6C). Strikingly, L1 retrotransposition was reduced by more than 50% in JFH1-SGR^BSD^ and in Con1-SGR cells compared to naïve Huh7.5 cells (Fig 6C), indicating that HCV RNA replication, the expression of the non-structural proteins of the replicase complex (NS3–NS5B), the formation of HCV replication compartments, or host-responses towards viral infection interfere with L1 retrotransposition. We next sought to distinguish if non-structural proteins or viral RNA are responsible for suppressing L1 mobilization. Ectopic expression of the HCV polyprotein from NS3–NS5B as well as plasmid-based expression of ^FLAG^core did not change L1 retrotransposition frequencies compared to control cells (Fig 6D), indicating that the decreased L1 retrotransposition in HCV-SGR cells is linked to presence or replication of HCV RNA. As the 3’ UTR of HCV contains a pathogen-associated molecular pattern that is sensed by HCV-infected cells [88,89], we transfected L1_RP_-FLuc-expressing cells with *in vitro*-transcribed HCV JFH1 3’ UTR RNA. Strikingly, we observed ~75% reduction in L1 retrotransposition frequencies compared to control cells (Fig 6E). This result suggests that HCV RNA-induced mechanisms restrict retrotransposition of engineered L1 reporter elements.

One peculiar observation was that HCV infection induced expression of endogenous L1, but it also drastically reduced the retrotransposition frequency of marked L1 reporter elements (Figs 2 and 3). Interestingly, endogenous but not ectopically expressed L1ORF1p protein levels were decreased in Huh7.5 cells stably expressing the HCV core protein, but not in NS5A expressing cells (S11 Fig). This reduction of endogenous L1ORF1p levels again inversely correlated with a slightly increased L1 retrotransposition frequency (Fig 6B). Our observation is consistent with the recently reported decrease in L1 retrotransposition frequency of engineered L1 retrotransposition reporter constructs as consequence of an increased expression of truncated or full-length L1ORF1p in *trans* [90]. Therefore, endogenous L1ORF1p levels in HCV-infected vs stable HCV core-expressing cells might contribute to restricted or enhanced mobilization of engineered L1 reporter elements, respectively.

### HCV-induced stress granule formation correlates with restriction of L1 retrotransposition

**Fig 7:**
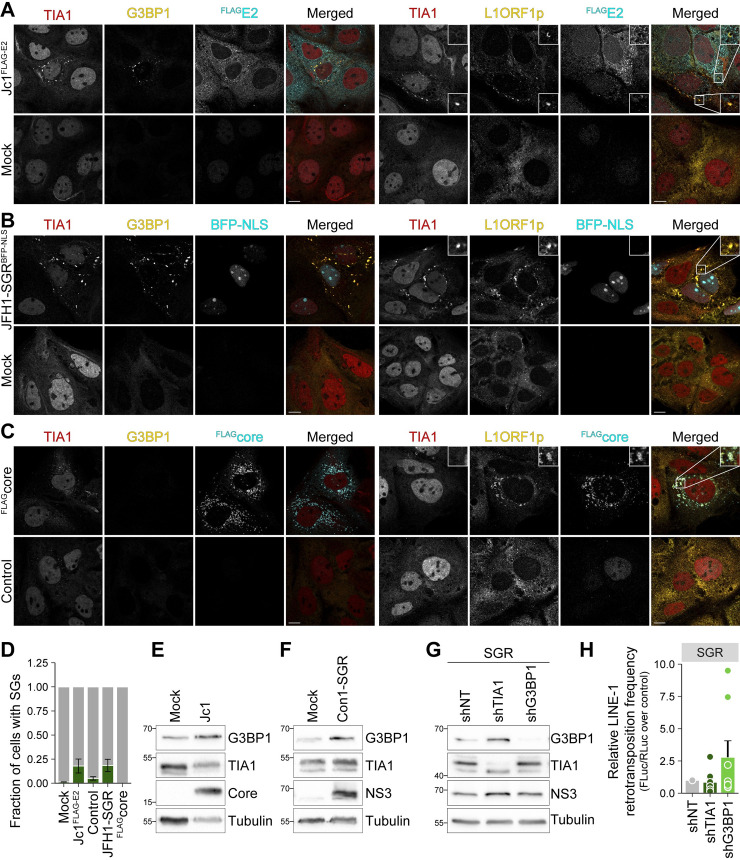
HCV RNA replication triggers the formation of stress granule which colocalize with L1ORF1p. (A–C) HCV RNA replication induces the formation of TIA1/G3BP1 positive stress granules. Confocal microscopy analysis of stress granule formation in HCV-infected and HCV replicon cells. Huh7.5 cells were electroporated with full-length HCV Jc1^FLAG-E2^ (A) or subgenomic JFH1-SGR^tagBFP-NLS^ (B) RNA; mock-electroporated cells were transfected with the ^FLAG^core expression plasmid or vector control (C). Cells were fixed at 3 days post electroporation and 2 days post transfection and stained with antibodies against TIA1, G3BP1, L1ORF1p, and FLAG as indicated. HCV infection was confirmed by staining of FLAG-E2 (A). The nuclear BFP signal marks HCV replicon positive cells (B). ^FLAG^core expression was visualized using a FLAG-specific antibody (C). Shown are representative images (scale bar 10 μm). (D) Quantification of stress granule (SGs) positive cells from (A–C). Fields were randomly selected and cells showingTIA1/G3BP1 positive granules were counted as SG positive (# of cells from 2 independent experiments: (A) mock = 142, Jc1^FLAG-E2^ = 130; (B and C) mock/vector control = 250, JFH1-SGR^tagBFP-NLS^ = 182, ^FLAG^core = 68)). (E–F) Immunoblot analysis of TIA1 and G3BP1 expression in HCV Jc1^NS5AB-EGFP^-infected Huh7.5 cells (E) and stable Huh7.5 Con1-SGR cells (F). Note that the immunoblot analyses presented in (F) were performed with the same samples as the immunoblots shown in Fig 2C. HCV core and NS3 served as markers for HCV infection and subgenomic replicons and tubulin was used as loading control. Shown are representative immunoblot analyses. (G) Knockdown of G3BP1 partially restores L1 retrotransposition frequency in HCV SGR cells. Stable Huh7.5 JFH1-SGR^BSD^ cells were transduced with lentiviral shRNAs targeting TIA1, G3BP1, or a non-targeting control (shNT). Knockdown efficiency was analyzed by immunoblotting using G3BP1- and TIA1-specific antibodies. HCV NS3 expression served as control for HCV replication and tubulin served as loading control. Shown is one representative immunoblot (n = 4). (H) shTIA1, shG3BP1, and shNT-transduced JFH1-SGR^BSD^ cells were transfected with the L1_RP_-FLuc reporter plasmid and luciferase assays were performed. Shown is the relative L1 retrotransposition frequency at 5 dpt of duplicate transfections from 4 independent experiments (mean ± SEM, n = 8).

Among the various mechanisms described to control L1 activity, it was reported that the SAM and HD domain containing protein 1 (SAMHD1) blocks L1 retrotransposition by promoting stress granule formation and enhancing sequestration of functional L1 RNPs and endogenous L1ORF1p in these stress granules [20]. As HCV RNA replication induces stress granule formation *in vitro* [54,57,91,92], we reasoned that L1 RNPs might be stalled in HCV-induced stress granules, hence not being able to complete the retrotransposition cycle. To explore this hypothesis, we performed immunofluorescence staining of HCV-infected cells for nucleolysin TIA-1 isoform p40/ T-cell-restricted intracellular antigen-1 (TIA1) and Ras GTPase-activating protein-binding protein 1 (G3BP1) as stress granule markers followed by confocal microscopy (Fig 7A–7C). HCV infection triggered cytoplasmic TIA1/G3BP1 positive stress granule formation in ~20% of HCV Jc1^FLAG-E2^-positive cells, whereas only 1.4% of HCV-negative cells displayed stress granules (Fig 7A and 7D). Likewise, ~18% of Huh7.5 cells transfected with HCV subgenomic replicon RNA (JFH1-SGR^tagBFP-NLS^) harbored TIA1/G3BP1 positive granules, compared to ~5% of control-electroporated cells (Fig 7B and 7D). In line with previous reports [57], the presence of HCV JFH1 3’ UTR RNA was sufficient to trigger stress granule formation (S13D Fig). Co-staining of endogenous L1ORF1p and TIA1 revealed localization of L1ORF1p to TIA1-positive granules and lipid droplets in Jc1^FLAG-E2^-positive cells, but only to TIA1-positive granules in JFH1-SGR^tagBFP-NLS^-positive cells (Fig 7A and 7B), indicating that L1ORF1p is indeed partially sequestered in stress granules. In contrast, ectopic expression of HCV core alone did not induce stress granule formation, although we observed a ring-like re-localization of L1ORF1p and to a lower extend a re-localization of TIA1 to HCV core–positive lipid droplets (Fig 7C and 7D). The redistribution of specific stress granule proteins to lipid droplets during HCV infection has been described by several groups [54,55,57,91]. Comparing our lipid droplet proteome dataset [53] to annotated stress granule and P-body proteins (downloaded from g:Profiler) [93] revealed that only a fraction of stress granule and P-body markers is found in lipid droplet fractions during HCV infection (S12 Fig), indicating that HCV infection alters and re-distributes stress granules. We next compared G3BP1 and TIA1 protein levels by immunoblot analysis and observed a pronounced increase in G3BP1 levels in HCV-infected Huh7.5 compared to the control (Figs 7E and S13A). G3BP1 levels were also slightly increased in Huh7.5-Con1-SGR cells compared to naïve Huh7.5 cells (Fig 7F), whereas stable expression of ^FLAG^core and NS5A^FLAG^ did not affect expression levels of the two stress granule markers TIA1 and G3BP1 (S13B Fig).

Finally, to mechanistically confirm our hypothesis that HCV restricts L1 retrotransposition by inducing stress granule formation, we transduced Huh7.5 JFH1-SGR^BSD^ cells with lentiviral shRNAs targeting G3BP1 and TIA1 and performed L1 retrotransposition assays. The selected shRNAs efficiently reduced G3BP1 and TIA1 protein levels in subgenomic replicon and in Huh7.5 cells (Figs 7G and S13C). Importantly, relative L1 activity was increased ~2.5 fold in G3BP1-knockdown cells compared to shNT control cells (Fig 7H). In contrast, knockdown of TIA1 did not restore L1 retrotransposition frequency. This might be due to the fact that downregulation of TIA1 concomitantly increased G3BP1 levels in JFH1-SGR^BSD^ cells (Fig 7G). In line, immunoblot analysis of HCV-infected cells showed increased G3BP1 level in TIA1-knockdown cells compared to shNT control cells and a complete failure of our shRNAs to reduce G3BP1 expression in HCV-infected cells (S13C Fig), suggesting compensatory upregulation of G3BP1 in HCV-infected cells. Taken together, our data indicate that L1 retrotransposition is restricted in HCV RNA-replicating cells due to sequestration of L1-encoded proteins in HCV RNA-induced stress granules.

### L1ORF1p expression does not affect HCV replication

**Fig 8:**
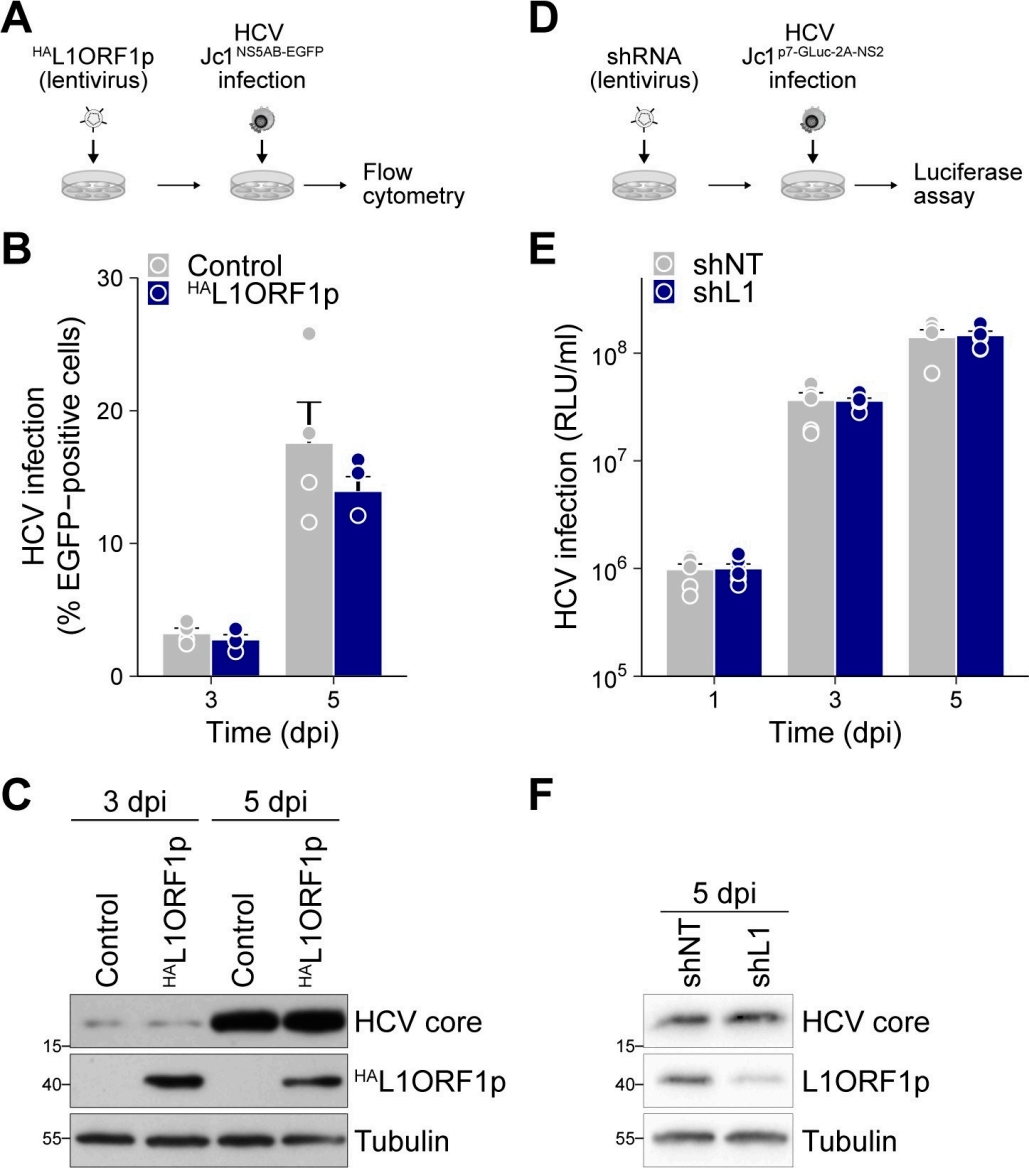
L1ORF1p does not affect HCV replication. (A) Experimental setup to test for the effect of L1ORF1p overexpression on HCV infectivity in Huh7.5 cells. ^HA^L1ORF1p was overexpressed by lentiviral transduction followed by infection with Jc1^NS5AB-EGFP^ (MOI 0.04). (B) HCV spreading kinetics were analyzed by flow cytometry of the EGFP reporter (mean ± SEM, n = 4). (C) Overexpression of ^HA^L1ORF1p was confirmed by immunoblot analysis. (D) Experimental setup to test for the effect of the downregulation of endogenous full-length L1 expression on HCV infectivity. Huh7.5 cells were transduced with shRNAs targeting the L1 5’ UTR (shL1) or a non-targeting control (shNT) for a minimum of 10 days prior to infection with the *Gaussia* luciferase (GLuc) reporter strain Jc1^p7-GLuc-2A-NS2^ (MOI 0.5). Luciferase reporter assays were performed to determine HCV infection kinetics. (E) Luciferase reporter assays to determine the effect of the knockdown of endogenous L1 expression on HCV replication. Results are shown as relative light units (RLU) per ml supernatant (mean ± SEM, n = 3). (F) Efficiency of L1 knockdown using shRNAs shL1 or shNT was confirmed by immunoblot analysis of endogenous L1ORF1p expression in cell lysates isolated at 5 dpi. Shown is one representative experiment.

As L1ORF1p is redistributed to HCV replication sites, we also assessed a potential function of L1ORF1p in HCV infection and replication by performing overexpression and knockdown experiments in Huh7.5 cells. As overexpressed ^HA^L1ORF1p resides in a complex with HCV RNA (Fig 5C), we analyzed the impact of ^HA^L1ORF1p overexpression on HCV infection. Huh7.5 cells stably expressing ^HA^L1ORF1p from a lentiviral vector were infected with a Jc1^NS5AB-EGFP^ reporter virus and analyzed for viral spreading by flow cytometry (Fig 8A). ^HA^L1ORF1p overexpression had no effect on HCV spreading (Fig 8B) despite strong overexpression of ^HA^L1ORF1p (Fig 8C). Conversely, we infected transduced cells expressing an shRNA targeting the L1 5’ UTR [94,95] or a non-targeting control with a Jc1^p7-GLuc-2A-NS2^ reporter virus [96] and determined GLuc activity as a measure for HCV infection kinetics (Fig 8D). Of note, the selected L1 shRNA (endo453) was described to perfectly match to the bidirectional promoter region of the 5’ UTR of functional L1Hs elements [94]. Again, we did not observe any changes in HCV infection rates (Fig 8E), regardless of the substantial downregulation of endogenous L1ORF1p protein levels (Fig 8F). Taken together, our data indicate that L1ORF1p does not function as host factor affecting HCV replication and its observed re-localization is rather a side effect than a requirement for a favorable HCV replication environment.

## Discussion

**Fig 9:**
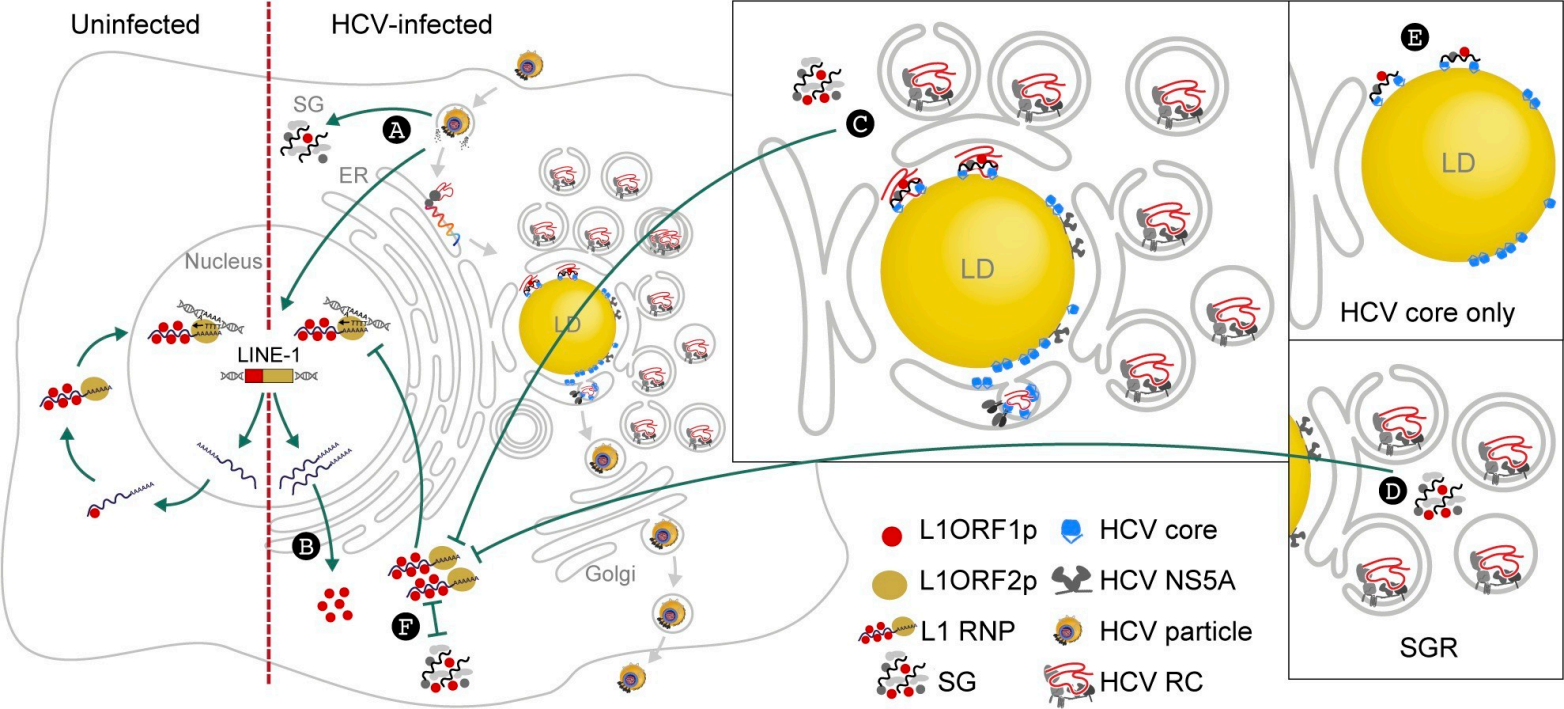
Model of HCV-L1 interaction and HCV-induced restriction of L1 retrotransposition. HCV infection (A) triggers stress granule (SG) formation, increases L1 expression and accumulation of L1ORF1p (B). In HCV-infected cells L1ORF1p is found in large cytoplasmic stress granules and, together with L1ORF1p-interacting proteins, is re-localized from cytoplasmic foci to lipid droplets. This re-localization depends on both HCV core trafficking to lipid droplets and the RNA-binding function of L1ORF1p. (C) Retrotransposition of engineered L1 elements is restricted in HCV-infected cells and in cells harboring HCV subgenomic replicons (SGR), which also show an increased stress granule formation (D). In contrast, ectopic expression of HCV core does not induce stress granules or impact L1 retrotransposition even though it redistributes L1ORF1p to lipid droplets (E). Thus, stress granule formation inversely correlates (⊢⊣) with L1 retrotransposition frequency, strongly suggesting that L1 RNPs are trapped within HCV-induced stress granules and not able to complete retrotransposition (F). HCV RC, HCV replication complex.

HCV profoundly reshapes the cellular landscape of infected cells in order to create advantageous conditions for viral RNA replication and virus production. In this context, we and others have observed the re-localization of various host proteins to HCV assembly sites [53–57], which are in close proximity to cytoplasmic lipid droplets [48,49]. Here, we followed up on previous results from a quantitative lipid droplet proteome analysis in which we identified L1ORF1-encoded peptides exclusively in lipid droplet fractions of HCV-infected hepatoma cells [53]. Based on our findings we suggest a model for the interaction between HCV and L1 explaining L1 retrotransposition inhibition in HCV-infected cells (Fig 9).

L1ORF1p is expressed from endogenous full-length L1 elements in uninfected Huh7.5 cells that represent a well-differentiated hepatocyte-derived cellular carcinoma cell line. We found that L1ORF1p accumulated in lipid droplet fractions isolated from HCV-infected Huh7.5 cells and from Huh7.5 cells expressing only the HCV core protein. HCV RNA replication was dispensable for L1ORF1p redistribution to lipid droplets but processing of HCV core, a prerequisite of core localization to lipid droplets [78], was required, indicating that L1ORF1p trafficking to lipid droplets is directly mediated by HCV core. Accordingly, L1ORF1p re-localized from cytoplasmic foci into half ring or ring-like structures at core-positive lipid droplets, often resembling the subcellular distribution of HCV core. However, in contrast to other RNA-binding and stress granule-associated proteins implicated in HCV replication and found at HCV assembly sites [53,55,57,82,91,97], L1ORF1p is not directly involved in HCV infection as demonstrated by knockdown and overexpression experiments. In order to investigate the effect of the overexpression of a full-length retrotransposition-competent L1 element on HCV infection, we transiently overexpressed the L1.3 retrotransposition reporter element from the pDK101 plasmid [17] in Huh7.5 cells. Unfortunately, overexpression of this L1 reporter had cytotoxic effects, and therefore, we were unable to assess HCV infection and replication in these cells. Comparing our total lipid droplet proteome dataset [53] with data obtained from a recent L1ORF1p interactome study [22], we identified 32 members of the lipid droplet proteome that interact with L1ORF1p. Strikingly, most L1ORF1p-interacting proteins enriched at lipid droplets during HCV infection are annotated RNA-binding proteins, suggesting a joint re-localization of L1ORF1p-associated RNP proteins. Together with L1ORF1p, MOV10 and PABPC1 were enriched in lipid droplet fractions from cells stably expressing HCV core but not from HCV NS5A-expressing cells. All three host genome-encoded proteins displayed an RNA-based interaction with HCV core, but no interaction with HCV NS5A, suggesting that HCV core is associated with L1ORF1p-containing RNPs and changes their subcellular localization. Consistently, the L1ORF1p RNA-binding mutant RR_261-262_AA [8,9,16] that does not localize to L1 RNPs/L1ORF1p foci [17,18], was not enriched in lipid droplet fractions upon HCV core expression. While we demonstrated an RNA-dependent HCV core-L1ORF1p interaction, there was no evidence for an association of L1ORF1p with NS5A although NS5A also harbors an RNA-binding domain [98]. However, NS5A has only been reported to bind the HCV genome, whereas HCV core was found to additionally associate with other RNAs *in vitro* [79–81,98]. Since HCV core, L1ORF1p, MOV10, and PABPC1 interact with each other independently of the presence of HCV RNA, we conclude that their interplay is not mediated by a joint binding of the viral genome, but likely by binding to those cellular RNAs that NS5A fails to bind.

Various host-defense mechanisms interfere with the amplification and overreaching activity of functional L1 elements in order to contain their mutagenic potential and maintain genome stability [35,68,99]. While the activity of L1 elements in human cancers has been studied more thoroughly [100], little is known about L1 activity in the context of exogenous viral infections. Using well-established engineered L1 retrotransposition reporter assays [66,67,101], we observed a strong decrease in L1 retrotransposition activity in acute and chronic HCV infection using cell-culture models. Our findings demonstrating that L1ORF1p interacts with HCV core and HCV infection restricts L1 mobilization, are reminiscent of a recent report showing that the HIV-1 Vpr protein interacts with L1ORF2p, thereby suppressing L1ORF2p reverse transcriptase activity and, as a consequence, restricting L1 retrotransposition [102]. On the contrary, it was previously reported that HIV-1 infection increases L1 retrotransposition frequency in an HIV-1 Vif- and Vpr-dependent manner, but the exact mechanism remains to be elucidated [103].

L1ORF1p is a nucleic acid chaperone that presumably facilitates the proposed nucleic acid remodeling steps involved in retrotransposition. Mutations that prevent binding of L1ORF1p to L1 mRNA and L1ORF1p localization to L1 RNPs abolish retrotransposition [9,16,17]. Initially, we hypothesized that the interaction of L1ORF1p with HCV core might alter L1 RNP integrity, thereby causing the observed decrease in L1 retrotransposition. Surprisingly, stable expression of HCV core alone had the opposite effect and slightly increased retrotransposition frequency of transiently transfected L1 reporter elements, demonstrating that HCV core-mediated L1ORF1p redistribution to lipid droplets does not cause the observed reduction of L1 retrotransposition frequency in HCV-infected cells. In contrast, cells harboring subgenomic HCV replicons (JFH1-SGR^BSD^ and Con1-SGR) that lack the structural proteins including core, as well as cells transfected with HCV 3’ UTR RNA, displayed reduced L1 retrotransposition frequencies similar to HCV-infected cells, indicating that presence of the HCV RNA impairs L1 retrotransposition.

L1 RNPs are known to associate with stress granules [18,19] and the enhanced sequestration of L1 RNPs in stress granules has been described to efficiently block L1 retrotransposition [20]. Further, artificial induction of stress granule formation increases L1ORF1p localization to TIA1-positive structures [104]. In line with previous publications [57,91,92,97], we observed a strong induction of the formation of G3BP1/TIA1-positive stress granules in HCV-infected and HCV-SGR cells. Concordantly, G3BP1 expression increased in HCV-infected Huh7.5 cells over time and was elevated, albeit to a lower extent, in Huh7.5-Con1-SGR cells. We detected L1ORF1p at lipid droplets as well as in large TIA1-positive granules in HCV-infected cells. This is in line with a previous report showing different subpopulations of granular structures in HCV-infected cells with small and disperse granules at lipid droplets and larger granules throughout the cytoplasm [57]. Even though stress granules seem to be altered and partially re-distributed as smaller granules to HCV replication sites at lipid droplets [54,57], L1ORF1p might be sequestered in both structures. Although we detected stress granules in only ~20% of HCV-positive cells, the oscillating nature of these dynamic structures [57,97] or predisposition of HCV-positive cells to form stress granules might explain the pronounced effect in limiting retrotransposition of engineered L1 reporter elements.

Knockdown of G3BP1, but not TIA1, partially rescued L1 retrotransposition in JFH1-SGR^BSD^ Huh7.5 cells. We were not able to rescue L1 retrotransposition by downregulation of G3BP1 or TIA1 in HCV-infected cells. However, HCV replication increased G3BP1 protein levels in TIA1-knockdown cells (both, infected and SGR cells) and induction of G3BP1 expression in HCV-infected cells restored G3BP1 protein level in G3BP1-knockdown cells, explaining the unsuccessful rescue of L1 retrotransposition under these conditions. In accordance with previous results [91], ectopic expression of HCV core did not trigger stress granule formation, although we sometimes observed a re-localization of TIA1 together with L1ORF1p to HCV core-positive areas upon strong HCV core expression. Taken together, our data indicate that HCV-induced stress granule formation leads to sequestration of L1ORF1p, which could be the major reason for the observed reduction of the L1 retrotransposition frequency in HCV-replicating cells.

In addition to the opposing effects of HCV core expression and HCV infection on engineered L1 retrotransposition and stress granule formation, we observed a transient increase of endogenous L1 mRNA levels and stably elevated L1ORF1p levels in HCV-infected cells, whereas the stable (lentivirus-driven) ectopic expression of HCV core decreased the amount of expressed L1ORF1p without affecting L1 mRNA expression levels. Recently, it was reported that transient or stable expression of full-length or truncated human L1ORF1 proteins suppresses L1 retrotransposition of marked L1 reporter elements in human cells [90]. Accordingly, the increased endogenous L1ORF1p level in HCV-infected cells might also contribute to the observed inhibition of engineered L1 retrotransposition in these cells, whereas lowered endogenous L1ORF1p levels in HCV core-expressing cells might account for increased L1 retrotransposition.

Despite its strong *cis* preference, the L1 protein machinery can occasionally mobilize *Alu* and SVA RNAs as well as host gene–encoded RNA polymerase II–transcribed mRNAs in *trans* [14,15,32,33,105–107]. There is evidence suggesting that both autonomous LTR retrotransposons (such as IAP) and autonomous non-LTR retrotransposons (such as human L1) can facilitate reverse transcription of non-retroviral RNA virus genomes into viral cDNAs as well as their subsequent genomic insertion [108–110]. Intriguingly, HCV DNA sequences were detected in genomic DNA isolated from mononuclear cells and liver biopsies of 4 out of 51 chronic HCV-infected patients [111] and the involvement of RT activity encoded by endogenous retroelements was considered. Our finding that HCV RNA was co-purified with ^HA^L1ORF1p suggests that the viral genome associates with an HCV core L1ORF1p RNP complex. However, we were not able to demonstrate reverse transcription of HCV RNA utilizing the L1 element amplification protocol (LEAP) [13]. Of note, the HCV genome lacks a 3’ poly(A) tract that is thought to be required for efficient L1-mediated mobilization *in trans* [112] and could explain why we were not able to identify rare events of L1ORF2p-mediated reverse transcription of HCV RNA. However, in chronically infected HCV patients, these events might still occur.

Hypomethylation of functional genomic L1 loci is often described as indicator for increased L1 expression and retrotransposition activity, and the latter has been addressed in numerous studies focusing on the role of L1 in cancer development [100]. Although HCV infection has been reported to induce persistent epigenetic changes [64], DNA methylation analysis of HCV-infected and mock-infected Huh7.5 cells did not reveal any differences in the methylation state of L1 5’ UTRs. However, our studies were limited to the hepatocellular carcinoma cell line Huh7.5, that displayed only partial CpG methylation of the L1 5’ UTR. Increased L1 mRNA expression as well as L1ORF1p expression were detected in several cancerous tissues and cell lines, including liver cancer [113,114], and the activity of functional L1 elements has been reported to be responsible for driving mutations in tumorigenesis and tumor progression [100]. As discussed above, we observed a reduced L1 retrotransposition frequency of engineered L1 reporter elements in HCV-replicating cells while endogenous L1ORF1p levels were stably elevated, indicating the expression of genomic protein-coding L1 loci. Interestingly, L1ORF1p expression alone was described to stimulate tumor cell proliferation [115,116]. Therefore, it is conceivable that the observed HCV-induced increase of endogenous L1ORF1p levels in HCV-infected cells might contribute to HCC progression in chronic HCV patients.

## Material and methods

### Reagents

The following antibodies, beads, and dyes were obtained commercially: HCV core (clone 7–50, sc-57800), HA (clone Y-11, sc-805-G), PABPC1 (clone 10E10, sc-32318), MOV10 (clone B-3, sc-515722), calnexin (c-20, sc-6465), TIA1 (c-20, sc-1751) (all Santa Cruz Biotechnology), G3BP1 (Clone 23/G3BP, 611127, BD Biosciences), PLIN2 (GP40, Progen; ab52356, abcam), HCV NS3 (ab65407, abcam), HCV NS5A (HCM-131-5, IBT), FLAG (F7425), FLAG (F1804), HA (H6908), tubulin (clone B5-1-2, T6074), anti-FLAG M2 affinity gel (A2220), anti-HA affinity gel (HA-7, A2095), recombinant protein A (15918–014) and protein G (15920–010) agarose beads (all Sigma), L1ORF1p (clone 4H1, MABC1152, Merck), YB-1 (ab12148, abcam), HRP-labelled secondary antibodies (Jackson ImmunoResearch), HRP-labelled TrueBlot secondary antibodies (Rockland Immunochemicals), Alexa488-, Alexa555-, and Alexa647-conjugated secondary antibodies (all donkey, IgG (H+L)), BODIPY493/503 (D-3922), BODIPY 655/676 (B-3932) (all Life Technologies), Hoechst33342 (Thermo Fisher). L1ORF1p antibody #984 was described previously [33]. Oligonucleotides and PCR primer were purchased from Sigma. Restriction enzymes for molecular cloning were obtained from NEB, other enzymes from Thermo Fisher. Unless stated otherwise, chemicals were purchased from Sigma or Applichem and cell culture reagents from Gibco/Thermo Fisher.

### Cell lines

Huh7 cells were provided by Ralf Bartenschlager, Huh7.5 cells [117] and Huh7.5.1 [118] cells were obtained from Apath, LLC, and HEK293T cells from the American Type Culture Collection. All cell lines were grown in DMEM supplemented with 10% FBS (Biochrom Superior or Gibco) under standard cell culture conditions. All plasmid transfection experiments were performed using FuGENE 6 (Promega). *In vitro* transcribed HCV JFH1 3’ UTR RNA was transfected using the *Trans*IT-mRNA Transfection Kit (Mirus Bio LLC). For transfection of HCV replicon RNA (Con1-SGR, JFH1-SGR^tagBFP-NLS^, JFH1-SGR^BSD^, or Jc1ΔE1E2^NS5AB-EGFP-BSD^) or full-length HCV RNA, cells were electroporated with *in vitro*-transcribed RNA as described [53]. Huh7.5-Con1-SGR cells were established by selection of Con1-SGR RNA-electroporated Huh7.5 cells with 1 mg/ml G418. Huh7.5 cells electroporated with the JFH1-SGR^BSD^ subgenomic replicon RNA were cultured in medium containing 10 μg/ml blasticidin.

### Plasmids

Schemes of plasmids and constructs are presented in S1 Fig. The following plasmids were described previously: HCV JFH1 wild-type [53,62], Jc1 wild-type (J6/JFH1 chimera) [60], Con1 subgenomic replicon [65], full length or envelope-deleted HCV Jc1 reporter strains encoding fluorescent proteins and selection markers between a duplicated NS5A-NS5B cleavage site (Jc1^NS5AB-EGFP^, Jc1^NS5ABmKO2^, Jc1ΔE1E2^NS5AB-EGFP-BSD^) [58], HCV Jc1^FLAG-E2^ [59], HCV Jc1^p7-GLuc-2A-NS2^ [53], lentiviral LeGO-iCer2 vectors encoding FLAG-tagged HCV JFH1 core or NS5A [53], LeGOCer2 [119], lentiviral vectors and expression plasmids encoding HCV core^WT^ and core^SPMT^ (genotype 1b), the lentiviral vector encoding a FLAG-tagged HCV core (genotype 1b) and the FLAG-tagged HCV core (genotype 2a) expression plasmid [51,120], lentiviral HCV RFP-NLS-IPS expression construct [121], the L1_RP_-FLuc dual-luciferase reporter plasmid pYX017 [66], and the EGFP-based L1 reporter (pLRE3-EF1-mEGFPI and pLRE3-EF1-mEGFP(Δintron)) [67].

The HCV JFH1 subgenomic replicon SGR^tagBFP-NLS^ (gt 2a) was constructed by replacing core-NS2 from pBR322 JFH1 [53] with a tagBFP-NLS marker generated through overlap extension PCR using MXS_TagBFP [122] as a template (primers: JFH1-5’NTR fw ACCTGCCCCTAATAGGGGCGA, core-tagBFP rev CTCCTTAATCAGCTCGCTCATGGCGCGCCGGTTGGTGTTTCT, core-tagBFP fw AGAAACACCAACCGGCGCGCCATGAGCGAGCTGATTAAGGAG, BFPtag-NLS-P2A rev GAAGTTTGTGGCGCCGCTGCCGCCAACTTTTCTTTTCTTTTTTGGCATGTATCTGGCCACTGCCACCTC, P2A-NS3 fw GGCAGCGGCGCCACAAACTTC, NS3 rev CCCAACGACGTGGCCCCTAGGGCAGAGCAC) and Age I and Avr II restriction sites. For JFH1-SGR^BSD^ (gt 2a), core–NS2 was replaced with a blasticidin resistance gene generated through overlap extension PCR using Jc1^NS5AB-mKO2-BSD^ [58] as a template (primers: JFH1-5’NTR fw ACCTGCCCCTAATAGGGGCGA, core-BSD rev TTGAGACAAAGGCTTGGCCATGGCGCGCCGGTTGGTGTTTCT, core-BSD fw AGAAACACCAACCGGCGCGCCATGGCCAAGCCTTTGTCTCAA, BSD-P2A rev GAAGTTTGTGGCGCCGCTGCCGCCCTCCCACACATAACCAGA, P2A-NS3 fw GGCAGCGGCGCCACAAACTTC, NS3 rev CCCAACGACGTGGCCCCTAGGGCAGAGCAC) and Age I and Avr II restriction sites.

For ectopic expression of the HCV JFH1 NS3-NS5B polyprotein, the MXS chaining Kit [122] was used to construct an MXS EF1α::MCS-bGHpA pGK::Neo^R^-bGHpA vector, using MluI, SalI and XhoI restriction sites (oligonucleotides: MCS sense CGCGTAACTCGAGACGTATGCGGCCGCGGCAGTACAGGATCCGGATACCCATACGACGTACCAGATTACGCTTGAG; MCS as TCGACTCAAGCGTAATCTGGTACGTCGTATGGGTATCCGGATCCTGTACTGCCGCGGCCGCATACGTCTCGAGTTA). JFH1 NS3-NS5B was amplified from pBR322 JFH1 [53] (primers: LIC NotI HCV NS3 fw CGCTGTCGAGACGTATGCGGCCATG GCTCCCATCACTGCTTATG, LIC BamHI HCV_NS5B rev ACGTCGTATGGGTATCCGGATC CTACCGAGCGGGGAGTAGG) and cloned into the MXS EF1α::MCS-bGHpA pGK::Neo^R^ -bGHpA vector by ligation independent cloning using BamHI and NotI restriction sites.

Lentiviral shRNA constructs were cloned into pSicoR-MS1 as described [51,67] using the following target sequences: shL1 CCAGGCTTGCTTAGGTAAACA (endo 453 described in [94,95]), shTIA1 TCCTGGCTCATCTCTTTATTC, shG3BP1 TTAGTCTTTCACTTCCAATTT, shNT GCGCGATAGCGCTAATAATT. For the pEF1α-^HA^L1ORF1p expression vector L1ORF1 was cloned into pEBB [123] using CMV L1-RP [32] as a template and the BamHI restriction site (primers: pEBB^HA^L1ORF1 fw GATAGGATCCGCTAGCATGTACCCATACGATGTTCCAGATTACGCTCTCGAGATGGGGAAAAAACAGAAC, pEBB^HA^L1ORF1 rev GATAGGATCCTTACATTTTGGCATGATTTTG (gift from S. Wissing)). For stable expression, ^HA^L1ORF1p^WT^ and ^HA^L1ORF1p^Mut^ were cloned into the lentiviral pSicoR-MS1 lacking the U6 promoter (pSicoR-MS1 ΔU6) by overlap extension PCR using pEF1α-^HA^L1ORF1p and pSicoR-MS1 as template and NheI and EcoRI restriction sites (primers: ^HA^L1ORF1p fw TGGATCCGCTAGCATGTACCCATACGAT, L1ORF1p AAA rev CTCTGCTGCGGCTTGTAGGGTTTCTGCCGAGAG, AAA fw CAAGCCGCAGCAGAGTGGGGGCCAATATTC, ^HA^L1ORF1p-2A-rev TCGACGTCTCCCGCAAGCTTAAGAAGGTCAAAATTCATTTTGGCATGATT, 2A-mCherry fw TGCGGGAGACGTCGAGTCCAACCCTGGGCCAGTGAGCAAGGGCGAG, EcoRI-mCherry rev CTCGACGAATTCTTACTTGTACAG).

### Immunofluorescence and confocal microscopy

For immunofluorescence analysis, cells seeded on coverslips were fixed in 4% PFA, permeabilized 5 min in 0.1% Triton-X-100/PBS and incubated in blocking solution (5% BSA, 1% fish skin gelatin, 50 mM Tris in PBS). Following overnight incubation with primary antibodies in blocking solution, cells were incubated with Alexa Fluor-coupled secondary antibodies, lipid droplets were stained with BODIPY493/503 or BODIPY655/676, and coverslips were embedded in Mowiol mounting media. Confocal microscopy was performed on a Nikon C2+ or on a Leica TCS SP5 II confocal laser scanning microscope. For colocalization analysis, individual cells were analyzed using the Coloc2 function of Fiji [124] to calculate the Manders’ colocalization coefficient (MCC) and the Pearson’s correlation coefficient (PCC).

### Immunoblot and co-immunoprecipitation

Cells were lysed in NP-40 lysis buffer (50 mM Tris, pH 7.4, 150 mM NaCl, 1% Nonidet-P40) supplemented with 1 mM phenylmethylsulfonyl fluoride (PMSF) and 1x protease inhibitor cocktail (Sigma) for 30–60 min. Nuclei and cell debris were removed by centrifugation. Clarified lysates were subjected to SDS-PAGE followed by blotting onto a nitrocellulose membrane (GE Healthcare). In general, samples were run on the same gel, transferred to one membrane and probed with the respective antibodies. Bands were detected by chemiluminescence using Lumi-Light substrate (Roche), SuperSignal West Femto (Thermo Fisher), and ECL hyperfilm (Amersham) or Image Lab (BioRad). Band signal intensities were quantified using the quantification function of Image Lab or the densitometric quantification function of Fiji [124].

For immunoprecipitation, cells were lysed in NP-40 lysis buffer as described above. For analysis of RNA-based interactions, lysates were pre-incubated with 100 μg/ml RNAse A (Thermo Fisher) or 100 U/ml RNaseOUT (Invitrogen) for 45 min at 4°C, rotating. Prior to immunoprecipitation, successful RNAse A treatment was confirmed on an agarose gel after RNA isolation using Tri Reagent (Sigma). To capture HA-tagged or FLAG-tagged proteins, lysates were incubated with anti-HA or anti-FLAG M2 affinity gel (Sigma) for 1 h at 4°C, rotating. Subsequently, beads were washed four times in cold NP-40 lysis buffer. Precipitated proteins were eluted in Laemmli buffer and analyzed by immunoblotting.

For immunoprecipitation of endogenous proteins, cells were lysed as described above. Prior to clarification, lysates were passed 8 x through a 23 G needle. Clarified lysates were precleared for 30 minutes at 4°C, rotating, using protein A or protein G agarose beads (Sigma). Incubation with the respective antibodies was performed overnight at 4°C, rotating. Equilibrated protein A (for rabbit antibodies) or protein G (for mouse antibodies) agarose beads were added and immunoprecipitation was performed for ~6 hours at 4°C, rotating. Samples were processed as described above and analyzed by immunoblotting. All immunoprecipitation steps for endogenous proteins were performed using 0.5% NP 40 lysis buffer (50 mM Tris, pH 7.4, 150 mM NaCl, 0.5% Nonidet-P40) in presence of 100 U/ml RNAseOUT (Invitrogen).

To quantify HCV RNA copy numbers in cellular and L1ORF1p-associated RNPs, immunoprecipitation was performed in presence of 100 U/ml RNAseOUT, followed by RNA isolation and quantitative RT-PCR, and immunoblotting of aliquots.

### Lipid droplet isolation and subcellular fractionation

Lipid droplets were isolated as previously described with minor modifications [125]. In brief, cells were harvested in cold PBS, resuspended in hypotonic sucrose buffer (0.25 M sucrose, 1 mM EDTA supplemented with 1 mM DTT, 1x protease inhibitor cocktail and 1 mM PMSF) and lysed mechanically in a Dounce homogenizer. Post-nuclear supernatants (PNS) were overlaid with isotonic potassium phosphate buffer (0.1 M potassium phosphate pH 7.4, 100 mM KCl, 1 mM EDTA, supplemented with 1 mM PMSF) and centrifugation was performed for 2 h at 100 000 x *g*, 4°C in an SW60 rotor (Beckman Coulter). Floating lipid droplets were harvested using a bent canula, and PNS and lipid droplet fractions were analyzed by immunoblotting.

For subcellular fractionation, lipid droplets were separated from the microsomal fraction by differential centrifugation as previously described with minor modifications [126,127]. Briefly, cells were resuspended in hypotonic sucrose buffer, lysed mechanically in a Dounce homogenizer, and the resulting cell homogenate was centrifuged twice for 5 min at 600 x *g*, 4°C to separate the nuclei. To separate the mitochondria, the post-nuclear supernatant (PNS fraction) was centrifuged twice for 10 min at 10 000 x *g*, 4°C. The supernatant was transferred to an ultracentrifugation tube and overlaid with isotonic potassium phosphate buffer. Ultracentrifugation was performed in an SW60 rotor (Beckman Coulter) for 1 h at 100 000 x *g*, 4°C. The floating lipid droplet fraction was harvested as described above. The pelleted microsomal fraction was resuspended in RIPA lysis buffer (50 mM Tris, pH 7.4, 1 mM EDTA, 150 mM NaCl, 1% NP-40, 0.5% sodium deoxycholate, 0.1% SDS) supplemented with 1x protease inhibitor cocktail, 100 mM PMSF, and 2% Triton-X-100 to ensure complete solubilization of the ER-retained HCV core^SPMT^ protein. For each fraction, equal amounts of total protein were subjected to immunoblot analysis.

### L1 retrotransposition reporter assays

L1 retrotransposition was analyzed by transient transfection of target cells with a functional L1 reporter element containing a retrotransposition reporter gene that is interrupted by an intron. The reporter gene is only functional after splicing of the intron and integration of a cDNA copy of the reporter gene–harboring L1 element into the genome, thus indicating a full cycle of L1 retrotransposition [16,66,101].

The luciferase-based L1 retrotransposition reporter assay was performed using the dual-luciferase reporter plasmid pYX017 [66]. The encoded L1_RP_ element under the control of a CAG promoter/enhancer element includes a firefly luciferase reporter cassette to quantify marked L1 retrotransposition events, and the plasmid backbone additionally encodes a *Renilla* luciferase for normalization of transfection rates. Huh7.5 cells were transfected with pYX017 and lysed 5–6 days post transfection (dpt) using 1x passive lysis buffer (Promega). Transfected cells were selected with puromycin, except for experiments with Jc1^NS5AB-EGFP^-infected Huh7.5 cells shown in Fig 3B that were performed without selection. Luciferase activity was determined with the Dual-Luciferase Reporter Assay System (Promega) using a Centro LB 960 luminometer (Berthold Technologies). Inactive L1 control reporter elements (pYX015) [66] yielded firefly luciferase values below the limit of detection thus validating the assay.

For flow cytometry-based retrotransposition analysis, Huh7.5 cells were transfected with the L1 reporter plasmid pLRE3-EF1-mEGFPI or pLRE3-EF1-mEGFP(Δintron), a transfection control plasmid lacking the intron in the EGFP reporter cassette [67]. At 6 dpt, cells were fixed in 2% PFA and analyzed for EGFP expression via flow cytometry on a BD LSR Fortessa (BD Bioscience). A Jc1^NS5AB-mKO2^ reporter virus was used to discriminate HCV-infected cells from mock-infected cells by mKO2 expression. To confirm EGFP expression as marker for retrotransposition, cells transfected with L1_LRE3_-EGFP were treated with 10 μM reverse transcriptase inhibitor abacavir that inhibits L1 retrotransposition [128] or DMSO as vehicle control 1 dpt. Treatment was renewed every other day. Cells were fixed in 2% PFA at 6 dpt and analyzed by flow cytometry. Flowjo (Treestar) was used for flow cytometry data analysis.

### HCV infection assays

For generation of HCV viral stocks, *in vitro*-transcribed HCV RNA was prepared and electroporated into Huh7.5 or Huh7.5.1 cells as described previously [51,53]. Viral titers (TCID_50_) were determined by serial dilution on Huh7.5 cells stably expressing the RFP-NLS-IPS HCV reporter [53,121]. To measure HCV spreading in ^HA^L1ORF1p-overexpressing Huh7.5 cells, lentiviral transduced cells were infected with Jc1^NS5AB-EGFP^ and analyzed at the indicated time points by flow cytometry with a BD LSR Fortessa (BD Bioscience). Data were analyzed with Flowjo (Treestar). To determine HCV infection rates in L1 knockdown cells, shRNA-transduced Huh7.5 cells were infected with Jc1^p7-GLuc-2A-NS2^ as described [127] and *Gaussia* luciferase activity in the supernatant was measured using the *Renilla* Luciferase Assay System (Promega) and a Centro LB 960 luminometer (Berthold Technologies).

### Lentivirus production

Lentiviral particles were produced using HEK293T cells as described before [53,129]. Lentiviral stocks were titrated on Huh7.5 cells. All lentiviral transductions were performed in medium containing 4 μg/ml polybrene.

### RNA isolation and quantitative RT-PCR

Total cellular RNA was isolated from cells, clarified cell lysates, or immunoprecipitation samples using Tri Reagent (Sigma). Glycogen (RNA grade, Thermo Fisher) was added as carrier to samples in which low RNA yield was expected. RNA isolated from cells and clarified lysates was treated with rDNAseI (DNA-free Kit, Invitrogen). RNA was reversely transcribed using Superscript III reverse transcriptase (Invitrogen), random hexamer primers (Qiagen), and RNAseOut (Invitrogen). Maxima SYBR Green Mastermix (Thermo Fisher) or the Luna Universal qPCR Master Mix (NEB) were used for qRT-PCR analysis on a 7500HT Fast Real-time PCR System or a StepOne Plus Real time PCR System (both Applied Biosystems). The following qRT-PCR primers were used: L1ORF1p fw TCAAAGGAAAGCCCATCAGACTA; L1ORF1p rev TTGGCCCCCACTCTCTTCT [130]; L1ORF2p fw GAGAGGATGCGGAGAAATAGGA; L1ORF2p rev GGATGGCTGGGTCAAATGGT [131]; HCV fw CGGGAGAGCCATAGTGG; HCV rev AGTACCACAAGGCCTTTCG [51]; 18s rRNA fw GTAACCCGTTGAACCCCATT; 18s rRNA rev CCATCCAATCGGTAGTAGCG; ADAR1 fw ATCAGCGGGCTGTTAGAATATG; ADAR1 rev AAACTCTCGGCCATTGATGAC; APOBEC3A fw TGGCATTGGAAGGCATAAGAC; APOBEC3A rev TTAGCCTGGTTGTGTAGAAAGC; APOBEC3B fw CGCCAGACCTACTTGTGCTAT; APOBEC3B rev CATTTGCAGCGCCTCCTTAT; APOBEC3C fw CTTGGTTCTGCGACGACATAC; APOBEC3C rev TCCTGGTAACATGGATACTGGAA; APOBEC3D fw CTTTCGAGGCCCGGTACTAC; APOBEC3D rev GTGATCTGGAAGCGCCTGTTA; APOBEC3F fw GGCCCGCGTGAAGATTATG; APOBEC3F rev GAGTGGTGCTTTACAACTTCCA; APOBEC3G fw GCATCGTGACCAGGAGTATGA; APOBEC3G rev GTCAGGGTAACCTTCGGGT [132]; TRIM5α fw CTGGAGATGCTGAGGCAGAAGC; TRIM5α rev GTCCAGGATGTCTCTCAGTTGC (Origene # HP2162777); APOBEC3H fw CCCGCCTGTACTACCACTGG; APOBEC3H rev GGGTTGAAGGAAAGCGGTTT [133].

### *In vitro* transcription of HCV JFH1 3’ UTR RNA

For *in vitro* transcription of the HCV JFH1 3’ UTR, PCR was performed using pBR322 JFH1 [53] as template (primers: T7 JFH1 3 UTR fw TAATACGACTCACTATAG AGCGGCACACACTAGGTAC, JFH1 3 UTR rev ACATGATCTGCAGAGAGACCA), adding a T7 promoter to the 3’ UTR sequence. The resulting PCR product served as template for *in vitro* transcription using the MEGAscript T7 Transcription Kit (Thermo Fisher).

### Extraction of DNA and quantitative real time PCR

To quantify L1-Fluc *de novo* insertions in cells that were subjected to the L1 dual-luciferase reporter assay, DNA was isolated using the QuickExtract DNA Extraction kit (Lucigen via Biosearch Technologies) according to the manufacturer’s instructions. Briefly, cells were harvested, counted, and equal cell numbers were used for DNA isolation. Samples were analyzed for the presence of marked L1-FLuc *de novo* insertions by quantitative real-time PCR (qRT-PCR) using the Luna Universal Probe qPCR Master Mix (NEB) and the following primers and probes [66]: FLuc exon-exon junction probe CTTCCCACCTGCCACC; FLuc fw GCAAAAGAAGCTACCGATCATACA; FLuc rev GAAGCTCTCGGGCACGAA. To compare plasmid transfection efficiencies, we performed conventional SYBR green qPCR using the Luna Universal qPCR Master Mix (NEB) and the following primers binding to the puromycin resistance gene (PuroR) or *Renilla* luciferase gene encoded by the L1 dual-luciferase reporter plasmid pYX017: RLuc fw GGCAAGAGCGGGAATGGCT; RLuc rev CAGTCGTGGCCCACAAAGAT; PuroR fw AGCAACAGATGGAAGGCCTC; PuroR rev GGCGCTGCCCAGACCCTT. Of note, the assay was validated using an inactive L1-FLuc dual reporter pYX015 [66] that cannot retrotranspose and consequently was undetectable using the spliced FLuc probe and primers.

### L1 promoter methylation analysis

To compare L1 promoter methylation, genomic DNA was isolated from HCV Jc1^NS5AB-EGFP^-infected and uninfected Huh7.5 cells at different time points using the Quick DNA Miniprep Kit (Zymo Research) according to the manufacturer’s instructions. DNA methylation was analyzed by methylation-sensitive restriction enzyme digestion and PCR with gene-specific primers (MSRE-PCR) using the OneStep qMethyl-PCR Kit (Zymo Research) according to the manufacturer’s protocol. The following primers were designed to amplify a 363 bp fragment previously used for bisulfite sequencing analysis of a constellation of L1 loci, which included both young Ta-1 and older subfamilies of the L1Hs/L1PA1 family such as Ta-0 due to the high degree of L1 sequence conservation [134]: CpG analysis fw AAGGGGTCAGGGAGTTCCCTT; CpG analysis rev TGTCTGTGCCCTGCCCCCA.

### Statistical analysis

For statistical analysis, we used R and RStudio [135,136]. Statistical analysis was performed using an unpaired two tailed t-test with unequal variance (Welch t-test), as indicated in the Figure legends. qRT-PCR data of HCV RNA copies in immunoprecipitated samples were tested for significance using the Mann-Whitney *U* test. Samples size (n) represents independent experiments if not stated otherwise in the Figure legends.

## Supporting information

S1 Fig Plasmids and viral reporter constructs used in this study. To generate the pEF1α-^HA^L1ORF1p expression vector, L1ORF1 was cloned into pEBB [123] using CMV L1-RP [32] as a template (gift from S. Wissing, Gladstone Institute of Virology and Immunology, University of California, San Francisco, CA, USA). ^HA^L1ORF1p^WT^ and ^HA^L1ORF1p^Mut^ were cloned into the lentiviral pSicoR-MS1 [137] lacking the U6 promoter (pSicoR-MS1 ΔU6) by overlap extension PCR using pEF1α-^HA^L1ORF1p and pSicoR-MS1 as template (this study). The following plasmids and reporter constructs have been described previously: L1_RP_-FLuc dual-luciferase reporter plasmid pYX017 [66], EGFP-based L1 reporter constructs (pLRE3-EF1-mEGFPI and pLRE3-EF1-mEGFP(Δintron)) [67], full-length or envelope-deleted HCV Jc1 reporter strains encoding fluorescent proteins and selection markers between a duplicated NS5A-NS5B cleavage site (Jc1^NS5AB-EGFP^, Jc1^NS5ABmKO2^, Jc1ΔE1E2^NS5AB-EGFP-BSD^) [58], Jc1^FLAG-E2^ [59], Jc1^p7-GLuc-2A-NS2^ [53], and the Con1 subgenomic replicon [65]. The JFH1 subgenomic replicon SGR^tagBFP-NLS^ (gt 2a) and the JFH1 subgenomic replicon SGR^BSD^ were constructed by replacing core-NS2 from pBR322 JFH1 (Rosch et al., 2016) with the tagBFP marker or a blasticidin resistance gene (this study). Lentiviral vectors and expression plasmids encoding HCV core^WT^ and core^SPMT^ (genotype 1b) [51], lentiviral LeGO-iCer2 vectors encoding FLAG-tagged HCV JFH1 core or NS5A [53], LeGOCer2 [119], and the FLAG-tagged HCV core (genotype 1b and 2a) expression plasmids [51,120] have been described before. BSD, blasticidin-S deaminase; CAG, CAG promoter; Cer, cerulean; CMV, cytomegalovirus promoter; EF1α, elongation factor 1-alpha promoter; EGFP, enhanced green fluorescent protein; FLuc, firefly luciferase; GLuc, *Gaussia* luciferase; gt, genotype; IRES, internal ribosomal entry site; LTR, long terminal repeat; NLS, nuclear localization sequence; NeoR, neomycin resistance; NS, non-structural; ORF, open reading frame; PuroR, puromycin resistance; RLuc, *Renilla* luciferase; SA, splice acceptor; SD, splice donor; SFFV, spleen focus-forming virus promoter; tagBFP, blue fluorescent protein; Ub, ubiquitin promoter; UTR, untranslated region; XFP, fluorescent protein.
(TIF)

S2 Fig Colocalization analysis of L1ORF1p, lipid droplets and HCV core using Manders’ colocalization coefficients. (A) Colocalization analysis of endogenous L1ORF1p and lipid droplets from Fig 1E using Manders’ colocalization coefficients (MCC) M1 and M2. (# of cells from 2 independent experiments: mock = 119, Jc1^FLAG-E2^ = 109; mean ± SEM, ***p< 0.001, Welch’s *t*-test). (B) Colocalization analysis of overexpressed ^HA^L1ORF1p and lipid droplets (upper panel) or ^HA^L1ORF1p and HCV core (lower panel) from Fig 1F using Manders’ colocalization coefficients (MCC) M1 and M2. (# of cells 2 independent experiments: mock = 42, Jc1 = 26, JFH1 = 52; mean ± SEM, *p< 0.05, **p< 0.01, ***p< 0.001, Welch’s *t*-test).
(TIF)

S3 Fig HCV infection of hepatoma cells does not affect L1 promoter methylation. Time course analysis of the methylation status of CpG islands in the 5’ UTR of intact members of the L1 subfamily Ta-1 and of older subfamilies of the L1Hs/L1PA1 family in mock and Jc1^NS5AB-EGFP^-infected Huh7.5 cells (MOI = 0.2). Genomic DNA was isolated at 3, 6, 9 and more than 21 dpi. 5’ UTR CpG methylation levels were determined by real time PCR using MSRE-PCR. Human methylated and non-methylated standards served as control. Shown is the % of L1 5’ UTR CpG methylation (mean ± SEM, n _3–9 dpi_ = 3; n_> 21 dpi_ = duplicate of one single experiment).
(TIF)

S4 Fig HCV infection suppresses L1 *de novo* integration. (A) Scheme of the experimental setup. Mock or Jc1^FLAG-E2^-infected Huh7.5 cells were transfected with the dual-luciferase L1_RP_ reporter plasmid at 4 dpi. The following day, transfected cells were split equally and re-seeded to perform genomic DNA extraction and luciferase assay analysis from the same transfection. Cells were harvested at 6 days post transfection (10 dpi). (B) Genomic L1-FLuc *de novo* insertions were quantified by qRT-PCR using an exon-exon junction-specific TaqMan fluorogenic probe with flanking primers FLuc fw and FLuc rev. To compare plasmid transfection levels, conventional qRT-PCR using SYBR green was performed, using primers targeting the puromycin resistance cassette (PuroR) or the *Renilla* luciferase gene (RLuc) encoded on the plasmid backbone. (C) Relative L1 retrotransposition frequencies at 6 dpt of duplicate transfections from 3 independent experiments (mean ± SEM, n = 6, ***p< 0.001, Welch’s *t*-test). (D) Relative L1 integration frequency was calculated using the 2^-ΔΔCT method and normalization to RLuc (left panel) or PuroR (right panel) at 6 dpt of duplicate transfections from 3 independent experiments (mean ± SEM, n = 6, ***p< 0.001, Welch’s *t*-test).
(TIF)

S5 Fig HCV infection suppresses L1 retrotransposition of an engineered L1-EGFP reporter element. (A) Scheme of the EGFP-based L1 retrotransposition reporter assay. In the pLRE3-EF1-mEGFPI retrotransposition reporter, the EGFP gene is in antisense orientation and interrupted by an intron in sense orientation flanked by splice donor (SD) and acceptor (SA) sites ensuring that EGFP is expressed only after splicing, reverse transcription, and integration. Therefore, the percentage of EGFP-positive cells is proportional to the number of L1-EGFP *de novo* retrotransposition events. As transfection control, the plasmid pLRE3-EF1-mEGFP(Δintron) was used that lacks the EGFP-interrupting intron. (B) Scheme of the experimental setup to investigate the effect of HCV infection on L1 retrotransposition. Following infection with Jc1^NS5AB-mKO2^ (MOI 0.005), Huh 7.5 cells were transfected with the pLRE3-EF1-mEGFPI reporter plasmid or pLRE3-EF1-mEGFP(Δintron) at 2 or 7 dpi. Cells were fixed 6 days post transfection and analyzed for EGFP expression by flow cytometry. The lower panel shows one representative flow cytometry plot for active retrotransposition at 8 dpi. (C) Quantification of (B). Shown are infected EGFP-positive cells as percent of mock-infected control (mean ± SEM, n = 3, * *p*< 0.05, ** *p*< 0.01, Welch’s *t*-test). (D) In order to validate EGFP expression as a measure for L1 retrotransposition, Huh7.5 cells transfected with pLRE3-EF1-mEGFPI were treated with the reverse transcriptase inhibitor Abacavir, that has been shown to inhibit L1 retrotransposition [128], and analyzed by flow cytometry at 6 days post transfection (dpt). Depicted is one representative flow cytometry plot that indicates that Abacavir reduces the percentage of EGFP-positive cells. (E) Quantification of flow cytometry data presented in (D). Number of EGFP-positive cells is presented in percent relative to DMSO control (mean ± SEM, n = 4, **p< 0.01, Welch’s *t*-test).
(TIF)

S6 Fig HCV replication does not induce L1-restricting ISGs such as APOBEC3 genes, ADAR1, TRIM5α, and MOV10. mRNA expression levels of genes coding for APOBEC3 protein family members, ADAR1, TRIM5α, and MOV10 were determined by qRT-PCR using specific primers in mock or Jc1 ^NS5AB-EGFP^-infected cells (11 dpi, MOI 0.05; mean ± SEM, n = 2) or Huh7.5-Con1-SGR cells (mean ± SEM, n = 2–3). Shown are mRNA expression levels relative to 18S rRNA levels.
(TIF)

S7 Fig L1ORF1p redistribution and interaction with HCV core is genotype-independent. (A) Scheme of the experimental setup. Huh7.5 cells were either electroporated with Con1 subgenomic replicon (Con1-SGR, gt 1b) RNA encoding NS3–NS5B (see B), or transduced with a lentiviral expression construct for HCV core (gt 1b, see C), and lipid droplets were isolated by sucrose density centrifugation. (B–C) Immunoblot analysis of lipid droplet fractions isolated from Huh7.5 cells electroporated with Con1-SGR (n = 3) (B), or transduced with lentiviral expression constructs for core or the respective empty vector control (n = 3) (C). Shown are representative experiments. Tubulin and PLIN2 served as loading controls for post-nuclear supernatants (PNS) and lipid droplets (LDs), respectively. (D) Association of HCV core with endogenous L1ORF1p is genotype-independent. Lysates of cells transduced with lentiviral ^FLAG^core were incubated either with RNaseOUT or RNase A followed by FLAG-specific immunoprecipitation and immunoblotting. Shown is one representative experiment (n = 2).
(TIF)

S8 Fig L1ORF1p localization to lipid droplets is coupled to HCV core trafficking. (A) Scheme of core wild-type (core^WT^) trafficking to lipid droplets (top panel) versus stalled trafficking of the core signal peptide mutant (core^SPMT^) (bottom panel). Trafficking to lipid droplets requires cleavage of the core signal peptide by the cellular signal peptide peptidase. Mutations in the cleavage site prevent processing and consequently core^SPMT^ is retained at the ER. (B–D) L1ORF1p does not traffic to lipid droplets in absence of core trafficking. Subcellular fractionation assay of core^WT^ and core^SPMT^-expressing cells. Huh7 cells were transduced with lentiviral expression constructs for HCV core^WT^ or core^SPMT^. Two days post transduction, cells were harvested and subcellular fractionation was performed. Fractions were analyzed for the presence of endogenous L1ORF1p, core^WT^, and core^SPMT^ by immunoblotting. Calnexin (CANX) and PLIN2 served as marker proteins and loading controls for MMs and LDs. Shown is one representative experiment (n = 3). PNS, post-nuclear supernatants; MMs, microsomal membranes; LDs, lipid droplets (B). Confocal microscopy of Huh7 cells that were co-transfected with plasmids expressing ^HA^L1ORF1p and core^WT^ or core^SPMT^, fixed 1 dpt, and stained using core and HA antibodies and BODIPY655/676 to visualize lipid droplets (scale bar 10 μm) (C). Colocalization analysis of (C). Shown are Manders’ colocalization coefficients (MCC) M1 and M2 and Pearson’s correlation coefficients (PCC) (# of cells from 2 independent experiments: WT = 67, SPMT = 59; mean ± SEM, **p< 0.01, ***p< 0.001, Welch’s *t*-test) (D).
(TIF)

S9 Fig Comparison of the lipid droplet proteome of HCV-infected cells with the L1ORF1p interactome. (A) Euler diagram of the overlap between the lipid droplet proteome dataset of HCV-infected cells from Rösch *et al*. [53] and the L1ORF1p interactome from Goodier *et al*. [22]. Of note, the lipid droplet-proteome dataset was re-analyzed to include all proteins that were identified via unique peptides in HCV-infected and uninfected cells in 3 out of 4 independent experiments. (B) Heatmap depicting all proteins in the overlap ordered according to enrichment in the lipid droplet fraction of HCV-infected vs. uninfected cells. Red indicates enrichment, blue indicates depletion, gray indicates NA.
(TIF)

S10 Fig HCV core redistributes L1ORF1p-associated RNPs to lipid droplets. (A) The L1ORF1p-interacting proteins MOV10 and PABPC1 are enriched in lipid droplets fractions of HCV core-expressing Huh7.5 cells. Lipid droplet fractions isolated from transduced Huh7.5 cells expressing ^FLAG^core or NS5A^FLAG^ were subjected to immunoblot analysis for the presence of L1ORF1p-interacting proteins PABPC1 and MOV10 (n = 3 for ^FLAG^core, n = 2 for NS5A^FLAG^). (B) Redistribution of L1ORF1p to lipid droplets requires an intact RNA-binding function. Huh7.5 cells were first transduced with lentiviral constructs for ^HA^L1ORF1p^WT^ or its RR_261-262_AA RNA-binding mutant (^HA^L1ORF1p^Mut^) and subsequently transduced with a lentiviral construct expressing HCV core (gt 1b). Lipid droplets were isolated and analyzed for the presence of ^HA^L1ORF1p and HCV core by immunoblotting. PNS, post-nuclear supernatants; LDs, lipid droplets. Shown is one representative experiment (n = 3).
(TIF)

S11 Fig L1ORF1p protein levels inversely correlate with the mobilization of L1 reporter elements. (A) HCV core expression decreases endogenous L1ORFp levels. ^FLAG^core, NS5A^FLAG^, or control lentivirus-transduced Huh7.5 cells were lysed at 5 and 10 dpt and endogenous L1ORF1p levels were analyzed by immunoblotting. Tubulin served as loading control. (B) Quantification of protein bands detected in (A) depicts the relative L1ORF1p levels normalized to tubulin (mean ± SEM, n = 4). (C) L1 mRNA levels were determined by qRT-PCR of ^FLAG^core, NS5A^FLAG^, or control lentivirus-transduced cells (mean ± SEM, n^FLAG^core_5 dpt_ = 4, n^FLAG^core_10 dpt_ = 3, nNS5A^FLAG^_5 dpt_ = 3, nNS5A^FLAG^_10 dpt_ = 2, *p< 0.05, Welch’s *t*-test). (D) Immunoblot analysis of Huh7.5 cells transduced with lentiviral constructs for the expression of ^HA^L1ORF1p and ^FLAG^core. Tubulin served as loading control. Shown is one representative experiment (n = 3).
(TIF)

S12 Fig Comparison of the lipid droplet proteome of HCV-infected cells with annotated stress granule and P-body proteins. (A) Euler diagram of the overlap between the lipid droplet proteome dataset of HCV-infected cells from Rösch et al. [53] and annotated stress granule and P-body proteins (Downloaded from g:Profiler) [93]. (B) Heatmap depicting all proteins in the overlap with the lipid droplet proteome ordered according to enrichment in the lipid droplet fraction of HCV-infected vs. uninfected cells. Red, blue and gray coloring indicates enrichment, depletion, and ‘not applicable’, respectively. Right panel denotes if proteins are classified as P-body or stress granule protein. PB, P-body; SG, stress granule.
(TIF)

S13 Fig HCV infection increases G3BP1 expression. (A) Time course of TIA1 and G3BP1 protein levels during HCV infection. Huh7.5 cells were infected with Jc1^NS5AB-EGFP^ (MOI = 0.2) or mock-infected, cells were lysed at the indicated time points and analyzed by immunoblotting using TIA1- and G3BP1-specific antibodies. HCV core expression was analyzed to confirm successful infection and tubulin served as loading control, respectively. For TIA1, the prominent upper band was quantified. Bar graph shows protein levels normalized to tubulin as fold over mock (mean ± SEM, n = 3–6). (B) Expression of HCV core and NS5A does not increase stress granule marker levels. Huh7.5 cells were transduced with lentiviral expression constructs for ^FLAG^core or NS5A^FLAG^ and stress granule protein levels were analyzed by immunoblotting using TIA1- and G3BP1-specific antibodies. Tubulin served as loading control. Shown is one representative experiment (n = 5). (C) HCV infection counteracts shRNA-mediated TIA1 and G3BP1 knockdowns. Huh7.5 cells were transduced with lentiviral shRNA constructs targeting TIA1, G3BP1, or a non-targeting control (shNT). Cells were infected with Jc1^NS5AB-EGFP^ or mock-infected and analyzed by immunoblot 13 dpi using TIA1- and G3BP1-specific antibodies. HCV core expression was analyzed to confirm successful infection and tubulin served as loading control (n = 3). (D) HCV 3‘ UTR RNA triggers stress granule formation. Huh7.5 cells were transfected with *in vitro* transcribed HCV JFH1 3‘ UTR RNA or mock-transfected and stained with G3BP1- and TIA1-specific antibodies and Hoechst. Shown are representative images (scale bar 10 μm). For quantification, fields were randomly selected and cells with TIA1/G3BP1-positive granules were counted as SG positive (# of cells from 2 independent experiments: n_3’ UTR_ = 96; n_Mock_ = 103).
(TIF)
