# Supplementary figures and images for: Hepatitis C virus infection restricts human LINE-1 retrotransposition in hepatoma cells
Source: PLoS Pathog. 2021 Apr 19;17(4):e1009496. doi: 10.1371/journal.ppat.1009496 (PMC8084336; doi:10.1371/journal.ppat.1009496)

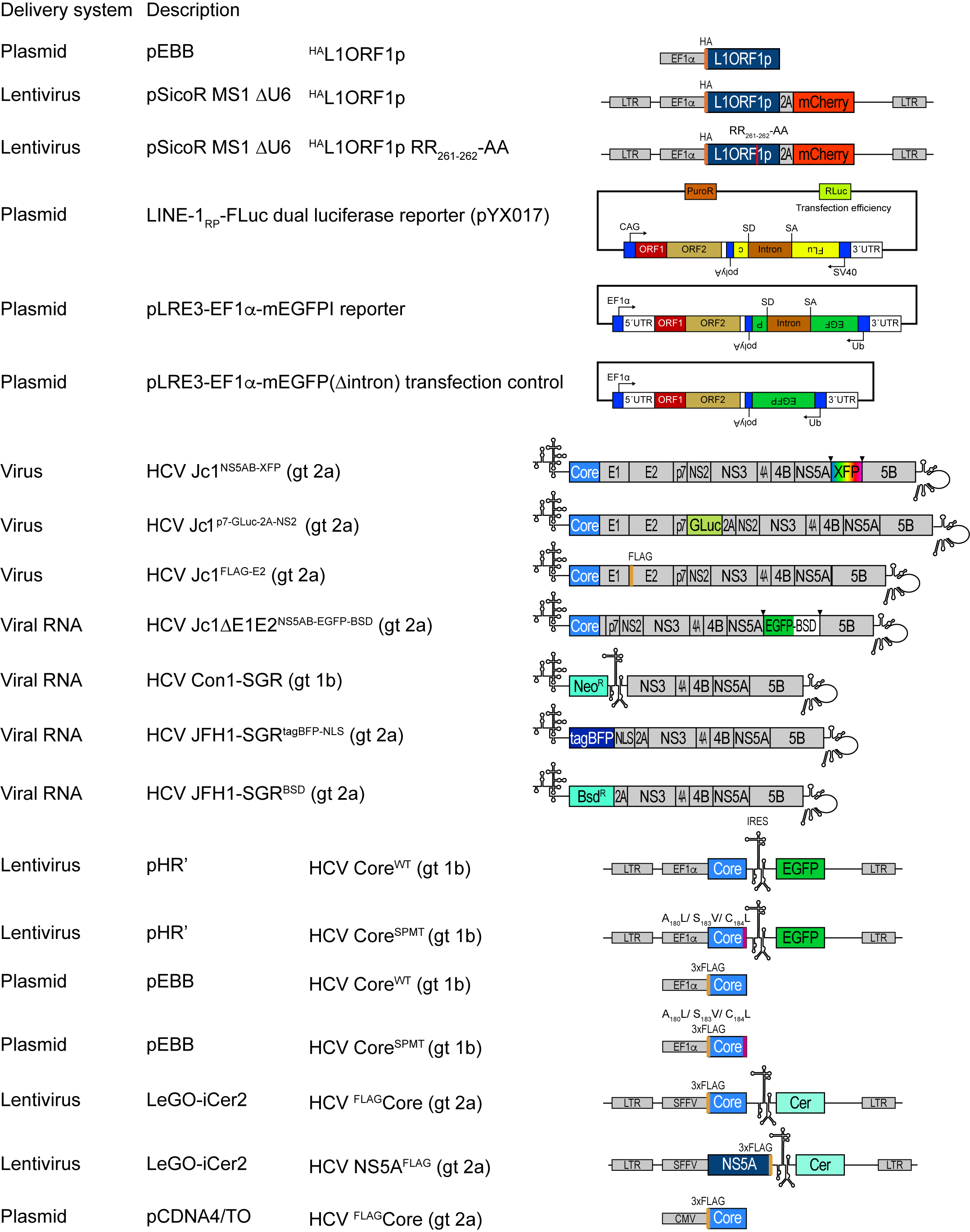

Supplement: S1 Fig — To generate the pEF1α-HAL1ORF1p expression vector, L1ORF1 was cloned into pEBB [123] using CMV L1-RP [32] as a template (gift from S. Wissing, Gladstone Institute of Virology and Immunology, University of California, San Francisco, CA, USA). HAL1ORF1pWT and HAL1ORF1pMut were cloned into the lentiviral pSicoR-MS1 [137] lacking the U6 promoter (pSicoR-MS1 ΔU6) by overlap extension PCR using pEF1α-HAL1ORF1p and pSicoR-MS1 as template (this study). The following plasmids and reporter constructs have been described previously: L1RP-FLuc dual-luciferase reporter plasmid pYX017 [66], EGFP-based L1 reporter constructs (pLRE3-EF1-mEGFPI and pLRE3-EF1-mEGFP(Δintron)) [67], full-length or envelope-deleted HCV Jc1 reporter strains encoding fluorescent proteins and selection markers between a duplicated NS5A-NS5B cleavage site (Jc1NS5AB-EGFP, Jc1NS5ABmKO2, Jc1ΔE1E2NS5AB-EGFP-BSD) [58], Jc1FLAG-E2 [59], Jc1p7-GLuc-2A-NS2 [53], and the Con1 subgenomic replicon [65]. The JFH1 subgenomic replicon SGRtagBFP-NLS (gt 2a) and the JFH1 subgenomic replicon SGRBSD were constructed by replacing core-NS2 from pBR322 JFH1 (Rosch et al., 2016) with the tagBFP marker or a blasticidin resistance gene (this study). Lentiviral vectors and expression plasmids encoding HCV coreWT and coreSPMT (genotype 1b) [51], lentiviral LeGO-iCer2 vectors encoding FLAG-tagged HCV JFH1 core or NS5A [53], LeGOCer2 [119], and the FLAG-tagged HCV core (genotype 1b and 2a) expression plasmids [51,120] have been described before. BSD, blasticidin-S deaminase; CAG, CAG promoter; Cer, cerulean; CMV, cytomegalovirus promoter; EF1α, elongation factor 1-alpha promoter; EGFP, enhanced green fluorescent protein; FLuc, firefly luciferase; GLuc, Gaussia luciferase; gt, genotype; IRES, internal ribosomal entry site; LTR, long terminal repeat; NLS, nuclear localization sequence; NeoR, neomycin resistance; NS, non-structural; ORF, open reading frame; PuroR, puromycin resistance; RLuc, Renilla luciferase; SA, splice acceptor; SD, s [file ppat.1009496.s001.tif]

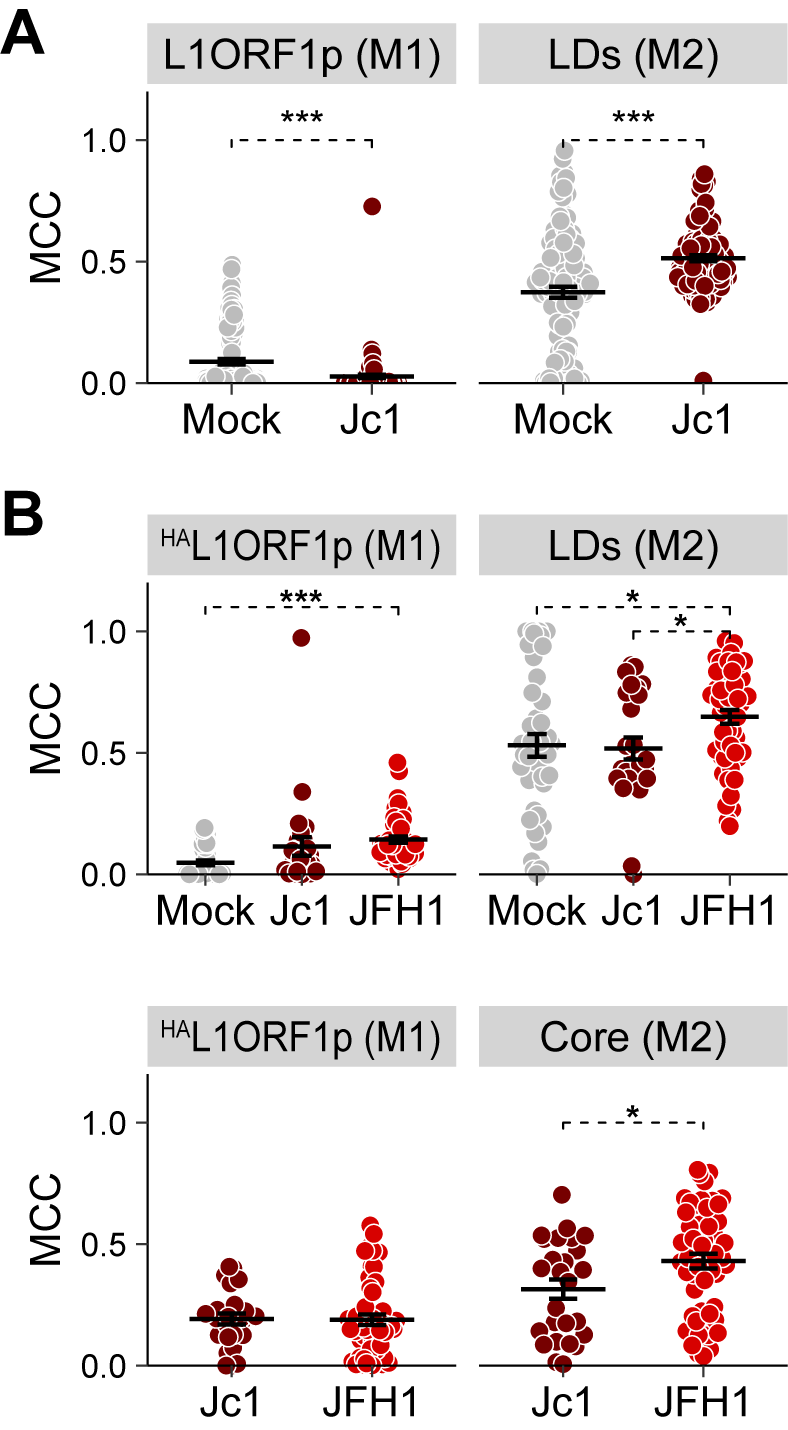

Supplement: S2 Fig — (A) Colocalization analysis of endogenous L1ORF1p and lipid droplets from Fig 1E using Manders’ colocalization coefficients (MCC) M1 and M2. (# of cells from 2 independent experiments: mock = 119, Jc1FLAG-E2 = 109; mean ± SEM, ***p< 0.001, Welch’s t-test). (B) Colocalization analysis of overexpressed HAL1ORF1p and lipid droplets (upper panel) or HAL1ORF1p and HCV core (lower panel) from Fig 1F using Manders’ colocalization coefficients (MCC) M1 and M2. (# of cells 2 independent experiments: mock = 42, Jc1 = 26, JFH1 = 52; mean ± SEM, *p< 0.05, **p< 0.01, ***p< 0.001, Welch’s t-test). (TIF) [file ppat.1009496.s002.tif]

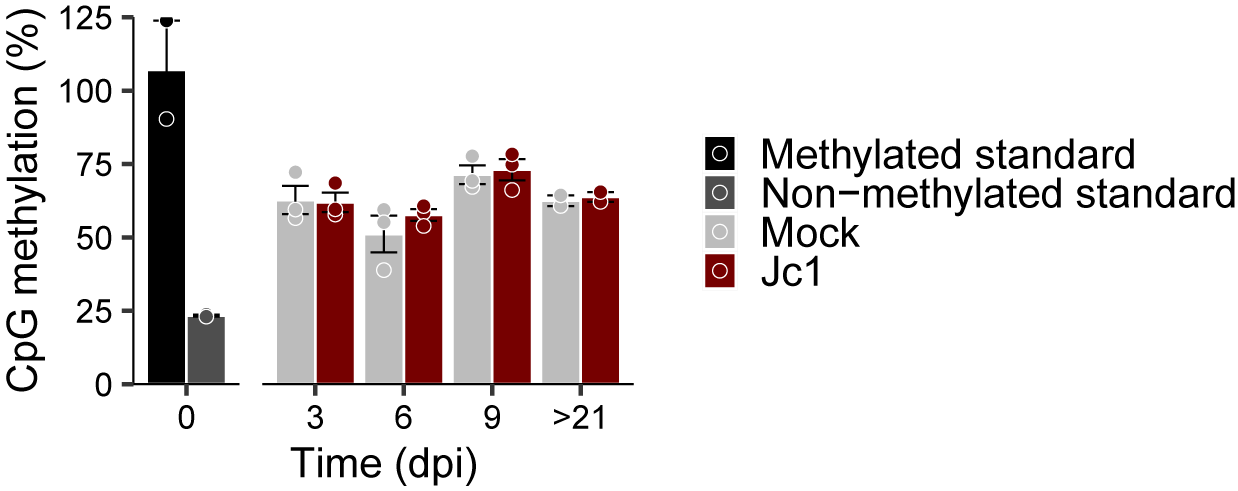

Supplement: S3 Fig — Time course analysis of the methylation status of CpG islands in the 5’ UTR of intact members of the L1 subfamily Ta-1 and of older subfamilies of the L1Hs/L1PA1 family in mock and Jc1NS5AB-EGFP-infected Huh7.5 cells (MOI = 0.2). Genomic DNA was isolated at 3, 6, 9 and more than 21 dpi. 5’ UTR CpG methylation levels were determined by real time PCR using MSRE-PCR. Human methylated and non-methylated standards served as control. Shown is the % of L1 5’ UTR CpG methylation (mean ± SEM, n 3–9 dpi = 3; n> 21 dpi = duplicate of one single experiment). (TIF) [file ppat.1009496.s003.tif]

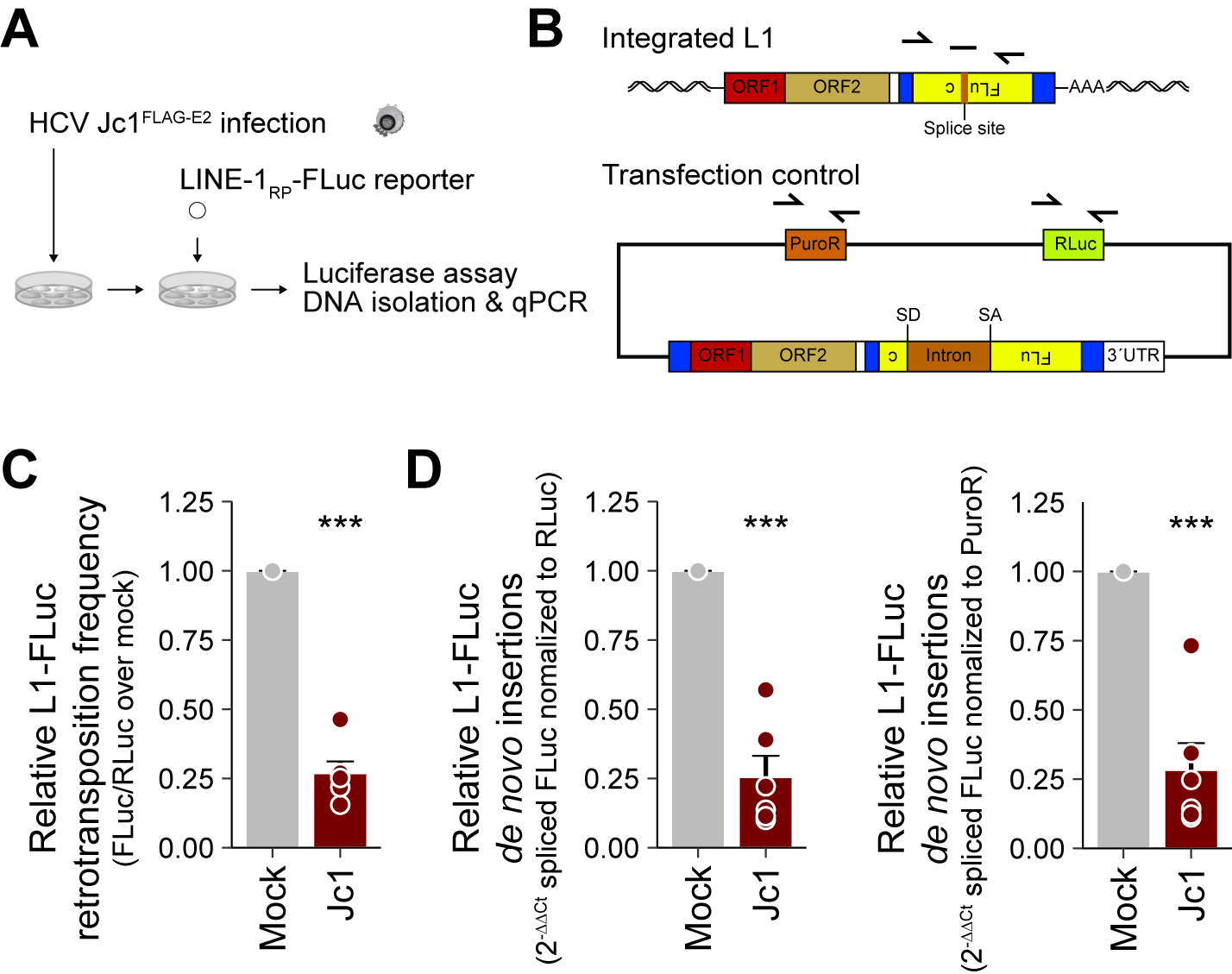

Supplement: S4 Fig — (A) Scheme of the experimental setup. Mock or Jc1FLAG-E2-infected Huh7.5 cells were transfected with the dual-luciferase L1RP reporter plasmid at 4 dpi. The following day, transfected cells were split equally and re-seeded to perform genomic DNA extraction and luciferase assay analysis from the same transfection. Cells were harvested at 6 days post transfection (10 dpi). (B) Genomic L1-FLuc de novo insertions were quantified by qRT-PCR using an exon-exon junction-specific TaqMan fluorogenic probe with flanking primers FLuc fw and FLuc rev. To compare plasmid transfection levels, conventional qRT-PCR using SYBR green was performed, using primers targeting the puromycin resistance cassette (PuroR) or the Renilla luciferase gene (RLuc) encoded on the plasmid backbone. (C) Relative L1 retrotransposition frequencies at 6 dpt of duplicate transfections from 3 independent experiments (mean ± SEM, n = 6, ***p< 0.001, Welch’s t-test). (D) Relative L1 integration frequency was calculated using the 2^-ΔΔCT method and normalization to RLuc (left panel) or PuroR (right panel) at 6 dpt of duplicate transfections from 3 independent experiments (mean ± SEM, n = 6, ***p< 0.001, Welch’s t-test). (TIF) [file ppat.1009496.s004.tif]

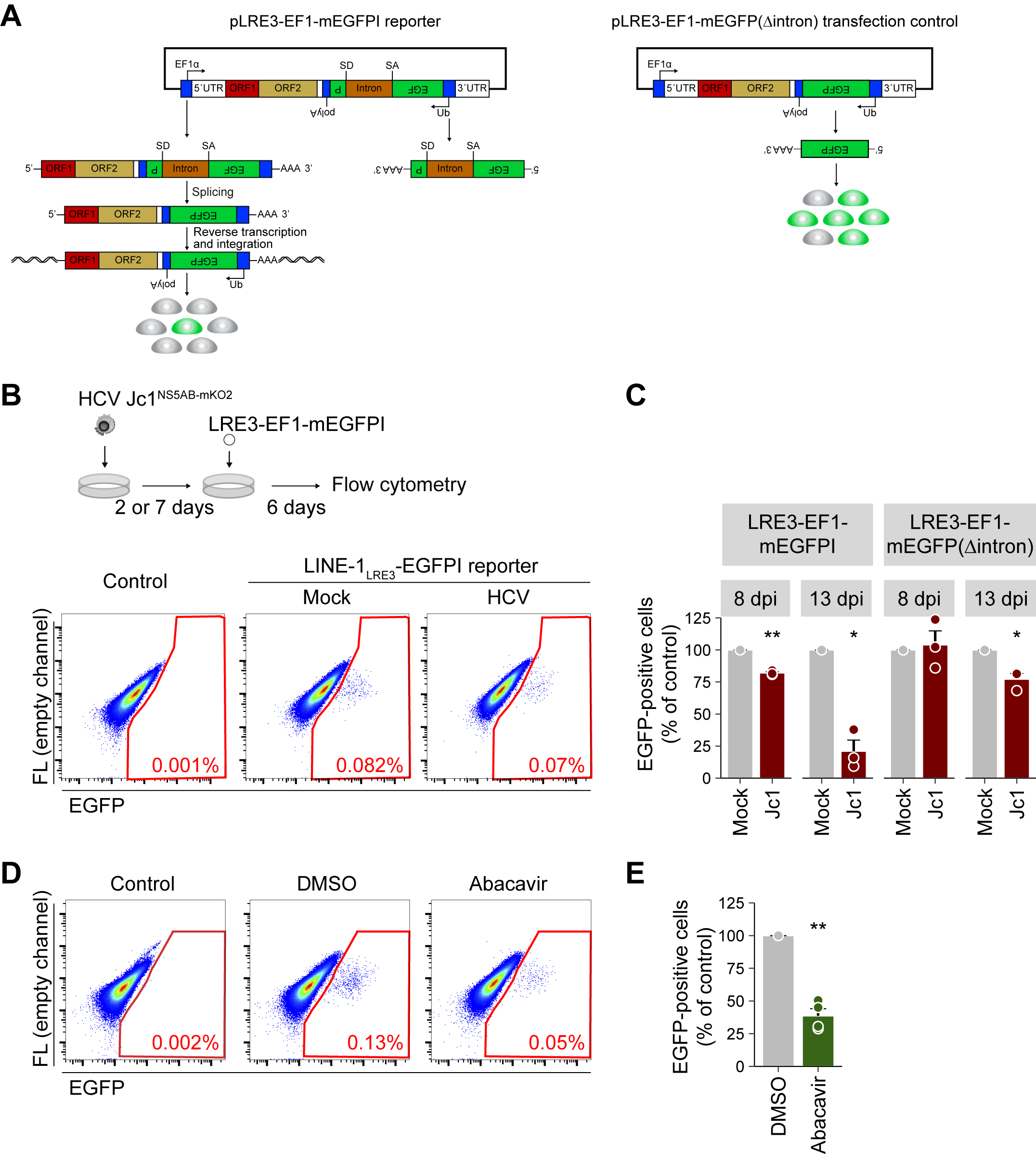

Supplement: S5 Fig — (A) Scheme of the EGFP-based L1 retrotransposition reporter assay. In the pLRE3-EF1-mEGFPI retrotransposition reporter, the EGFP gene is in antisense orientation and interrupted by an intron in sense orientation flanked by splice donor (SD) and acceptor (SA) sites ensuring that EGFP is expressed only after splicing, reverse transcription, and integration. Therefore, the percentage of EGFP-positive cells is proportional to the number of L1-EGFP de novo retrotransposition events. As transfection control, the plasmid pLRE3-EF1-mEGFP(Δintron) was used that lacks the EGFP-interrupting intron. (B) Scheme of the experimental setup to investigate the effect of HCV infection on L1 retrotransposition. Following infection with Jc1NS5AB-mKO2 (MOI 0.005), Huh 7.5 cells were transfected with the pLRE3-EF1-mEGFPI reporter plasmid or pLRE3-EF1-mEGFP(Δintron) at 2 or 7 dpi. Cells were fixed 6 days post transfection and analyzed for EGFP expression by flow cytometry. The lower panel shows one representative flow cytometry plot for active retrotransposition at 8 dpi. (C) Quantification of (B). Shown are infected EGFP-positive cells as percent of mock-infected control (mean ± SEM, n = 3, * p< 0.05, ** p< 0.01, Welch’s t-test). (D) In order to validate EGFP expression as a measure for L1 retrotransposition, Huh7.5 cells transfected with pLRE3-EF1-mEGFPI were treated with the reverse transcriptase inhibitor Abacavir, that has been shown to inhibit L1 retrotransposition [128], and analyzed by flow cytometry at 6 days post transfection (dpt). Depicted is one representative flow cytometry plot that indicates that Abacavir reduces the percentage of EGFP-positive cells. (E) Quantification of flow cytometry data presented in (D). Number of EGFP-positive cells is presented in percent relative to DMSO control (mean ± SEM, n = 4, **p< 0.01, Welch’s t-test). (TIF) [file ppat.1009496.s005.tif]

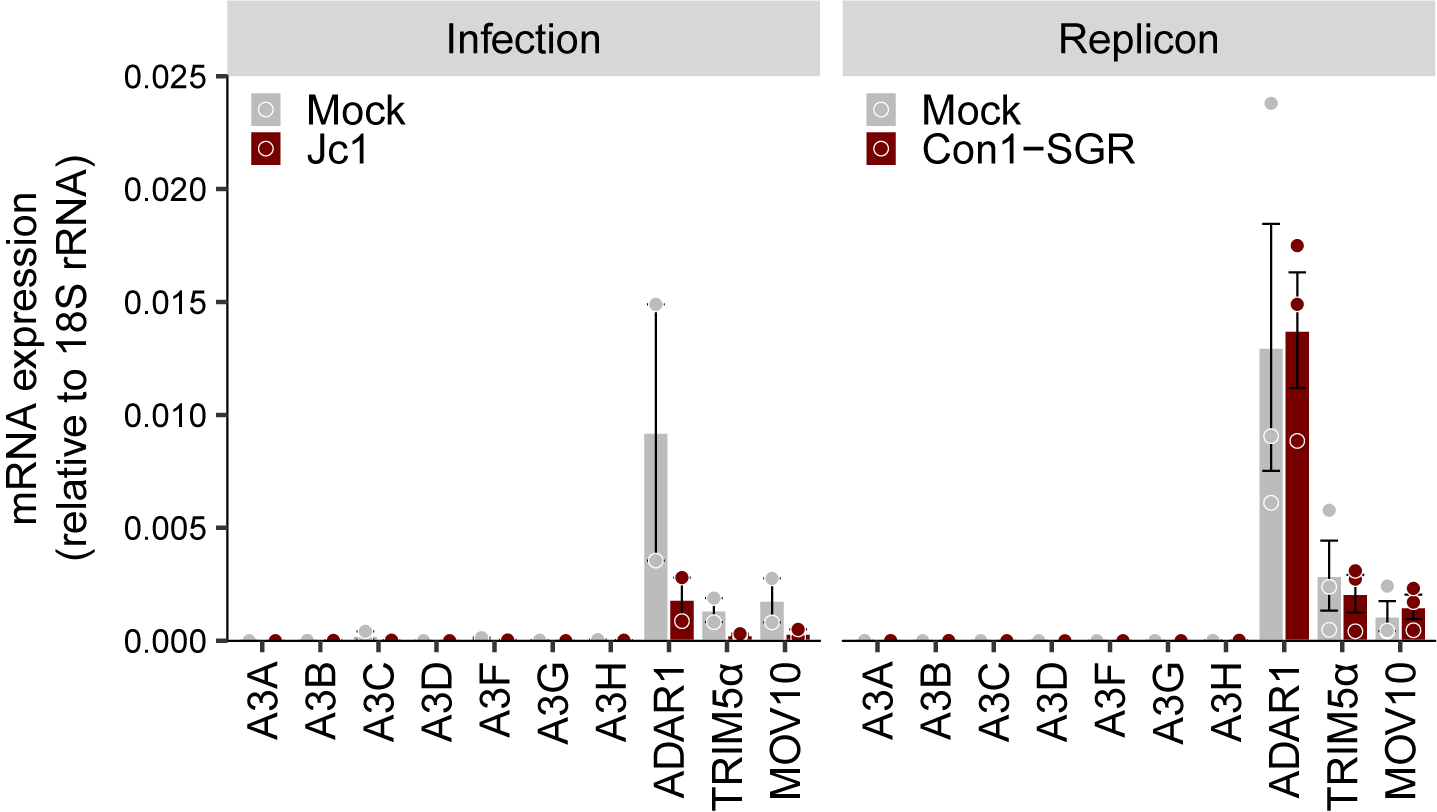

Supplement: S6 Fig — mRNA expression levels of genes coding for APOBEC3 protein family members, ADAR1, TRIM5α, and MOV10 were determined by qRT-PCR using specific primers in mock or Jc1 NS5AB-EGFP-infected cells (11 dpi, MOI 0.05; mean ± SEM, n = 2) or Huh7.5-Con1-SGR cells (mean ± SEM, n = 2–3). Shown are mRNA expression levels relative to 18S rRNA levels. (TIF) [file ppat.1009496.s006.tif]

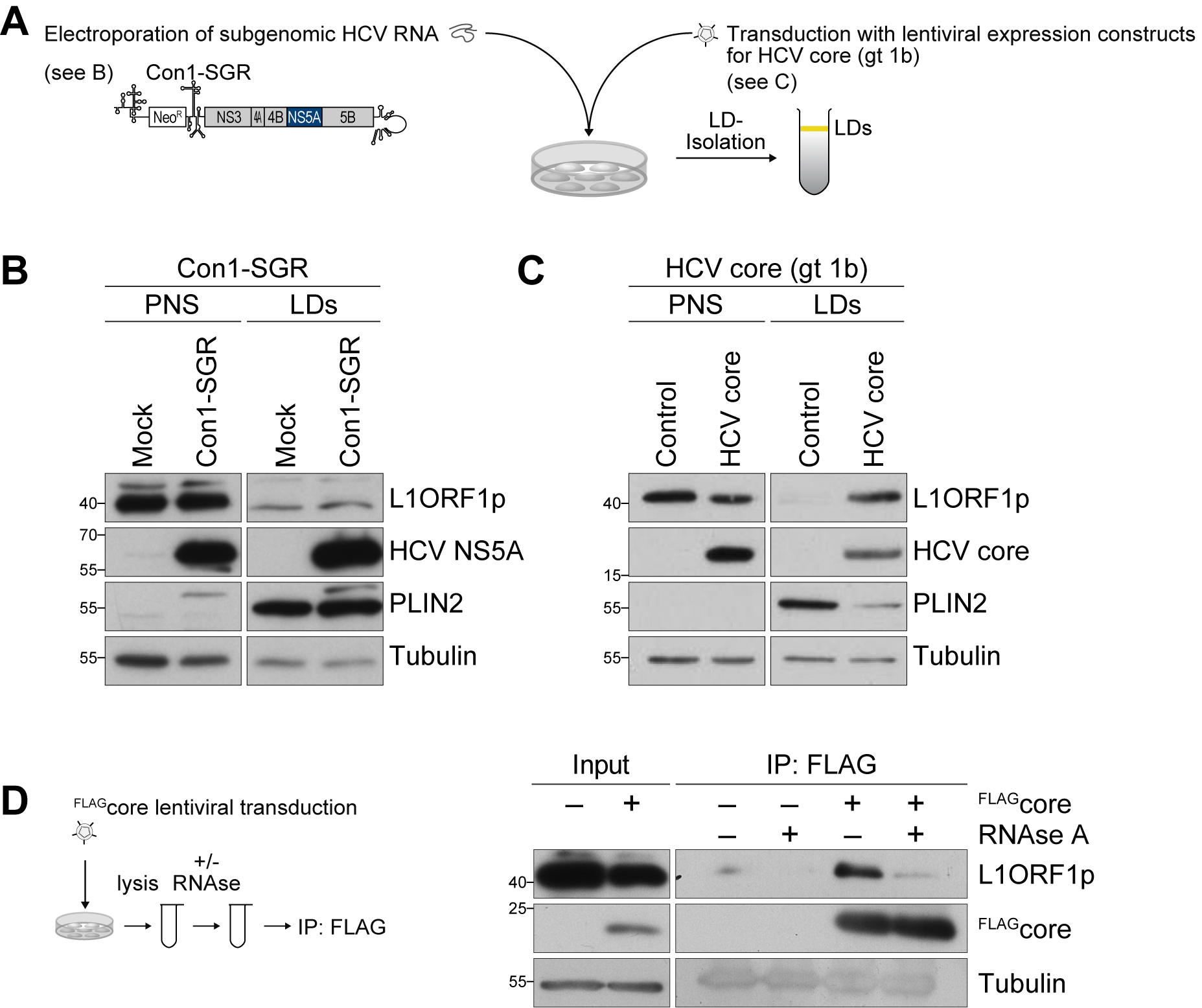

Supplement: S7 Fig — (A) Scheme of the experimental setup. Huh7.5 cells were either electroporated with Con1 subgenomic replicon (Con1-SGR, gt 1b) RNA encoding NS3–NS5B (see B), or transduced with a lentiviral expression construct for HCV core (gt 1b, see C), and lipid droplets were isolated by sucrose density centrifugation. (B–C) Immunoblot analysis of lipid droplet fractions isolated from Huh7.5 cells electroporated with Con1-SGR (n = 3) (B), or transduced with lentiviral expression constructs for core or the respective empty vector control (n = 3) (C). Shown are representative experiments. Tubulin and PLIN2 served as loading controls for post-nuclear supernatants (PNS) and lipid droplets (LDs), respectively. (D) Association of HCV core with endogenous L1ORF1p is genotype-independent. Lysates of cells transduced with lentiviral FLAGcore were incubated either with RNaseOUT or RNase A followed by FLAG-specific immunoprecipitation and immunoblotting. Shown is one representative experiment (n = 2). (TIF) [file ppat.1009496.s007.tif]

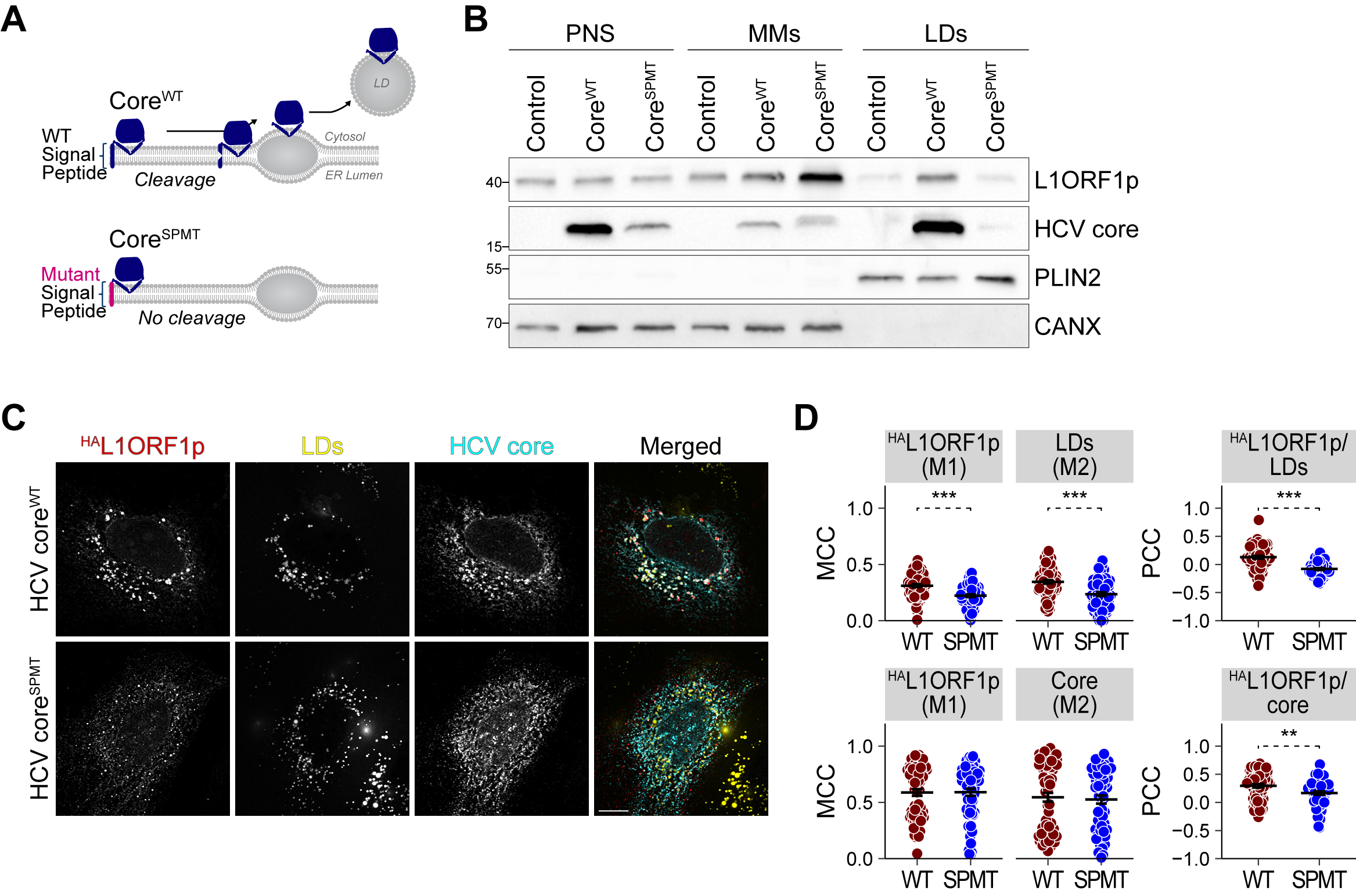

Supplement: S8 Fig — (A) Scheme of core wild-type (coreWT) trafficking to lipid droplets (top panel) versus stalled trafficking of the core signal peptide mutant (coreSPMT) (bottom panel). Trafficking to lipid droplets requires cleavage of the core signal peptide by the cellular signal peptide peptidase. Mutations in the cleavage site prevent processing and consequently coreSPMT is retained at the ER. (B–D) L1ORF1p does not traffic to lipid droplets in absence of core trafficking. Subcellular fractionation assay of coreWT and coreSPMT-expressing cells. Huh7 cells were transduced with lentiviral expression constructs for HCV coreWT or coreSPMT. Two days post transduction, cells were harvested and subcellular fractionation was performed. Fractions were analyzed for the presence of endogenous L1ORF1p, coreWT, and coreSPMT by immunoblotting. Calnexin (CANX) and PLIN2 served as marker proteins and loading controls for MMs and LDs. Shown is one representative experiment (n = 3). PNS, post-nuclear supernatants; MMs, microsomal membranes; LDs, lipid droplets (B). Confocal microscopy of Huh7 cells that were co-transfected with plasmids expressing HAL1ORF1p and coreWT or coreSPMT, fixed 1 dpt, and stained using core and HA antibodies and BODIPY655/676 to visualize lipid droplets (scale bar 10 μm) (C). Colocalization analysis of (C). Shown are Manders’ colocalization coefficients (MCC) M1 and M2 and Pearson’s correlation coefficients (PCC) (# of cells from 2 independent experiments: WT = 67, SPMT = 59; mean ± SEM, **p< 0.01, ***p< 0.001, Welch’s t-test) (D). (TIF) [file ppat.1009496.s008.tif]

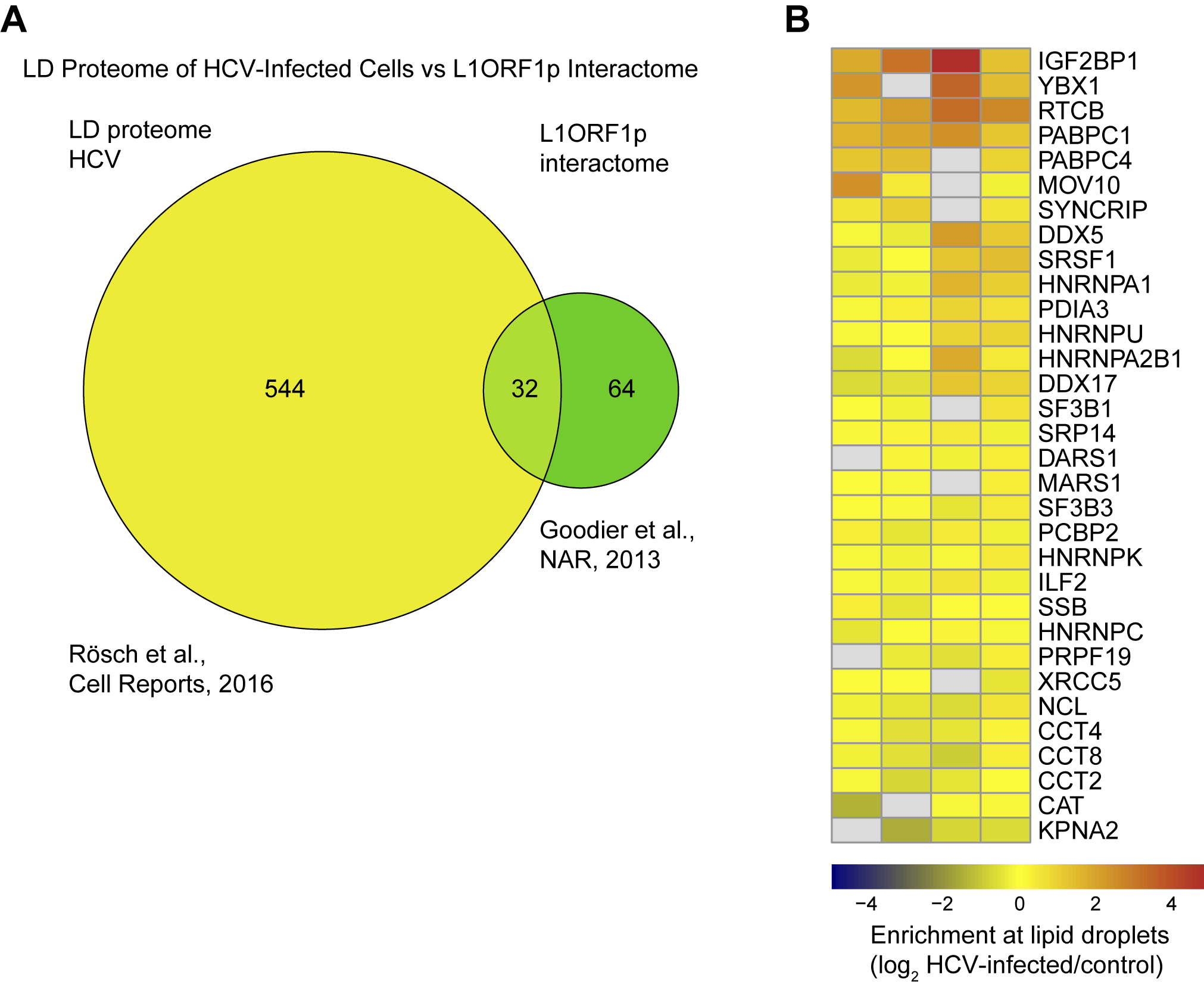

Supplement: S9 Fig — (A) Euler diagram of the overlap between the lipid droplet proteome dataset of HCV-infected cells from Rösch et al. [53] and the L1ORF1p interactome from Goodier et al. [22]. Of note, the lipid droplet-proteome dataset was re-analyzed to include all proteins that were identified via unique peptides in HCV-infected and uninfected cells in 3 out of 4 independent experiments. (B) Heatmap depicting all proteins in the overlap ordered according to enrichment in the lipid droplet fraction of HCV-infected vs. uninfected cells. Red indicates enrichment, blue indicates depletion, gray indicates NA. (TIF) [file ppat.1009496.s009.tif]

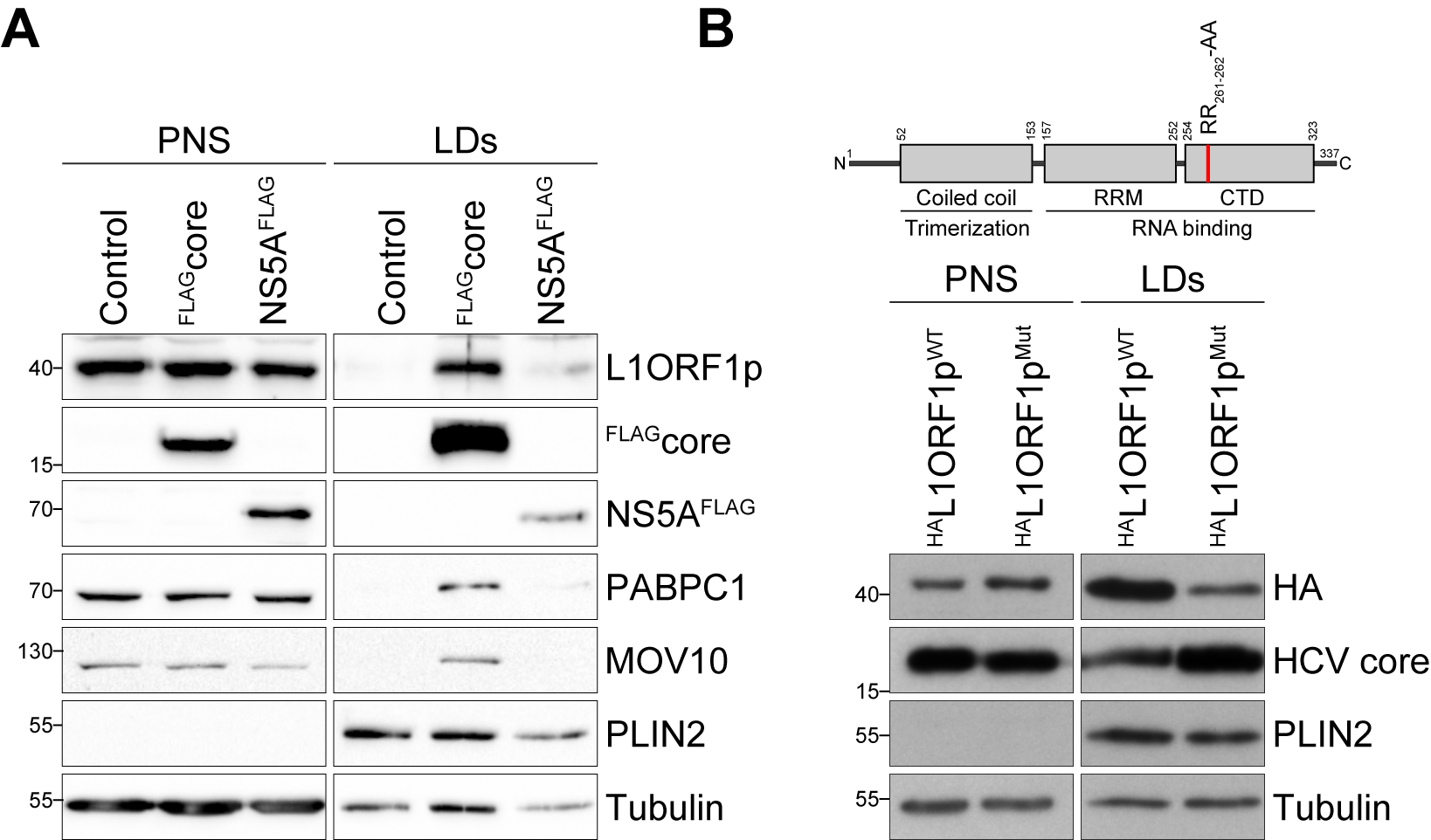

Supplement: S10 Fig — (A) The L1ORF1p-interacting proteins MOV10 and PABPC1 are enriched in lipid droplets fractions of HCV core-expressing Huh7.5 cells. Lipid droplet fractions isolated from transduced Huh7.5 cells expressing FLAGcore or NS5AFLAG were subjected to immunoblot analysis for the presence of L1ORF1p-interacting proteins PABPC1 and MOV10 (n = 3 for FLAGcore, n = 2 for NS5AFLAG). (B) Redistribution of L1ORF1p to lipid droplets requires an intact RNA-binding function. Huh7.5 cells were first transduced with lentiviral constructs for HAL1ORF1pWT or its RR261-262AA RNA-binding mutant (HAL1ORF1pMut) and subsequently transduced with a lentiviral construct expressing HCV core (gt 1b). Lipid droplets were isolated and analyzed for the presence of HAL1ORF1p and HCV core by immunoblotting. PNS, post-nuclear supernatants; LDs, lipid droplets. Shown is one representative experiment (n = 3). (TIF) [file ppat.1009496.s010.tif]

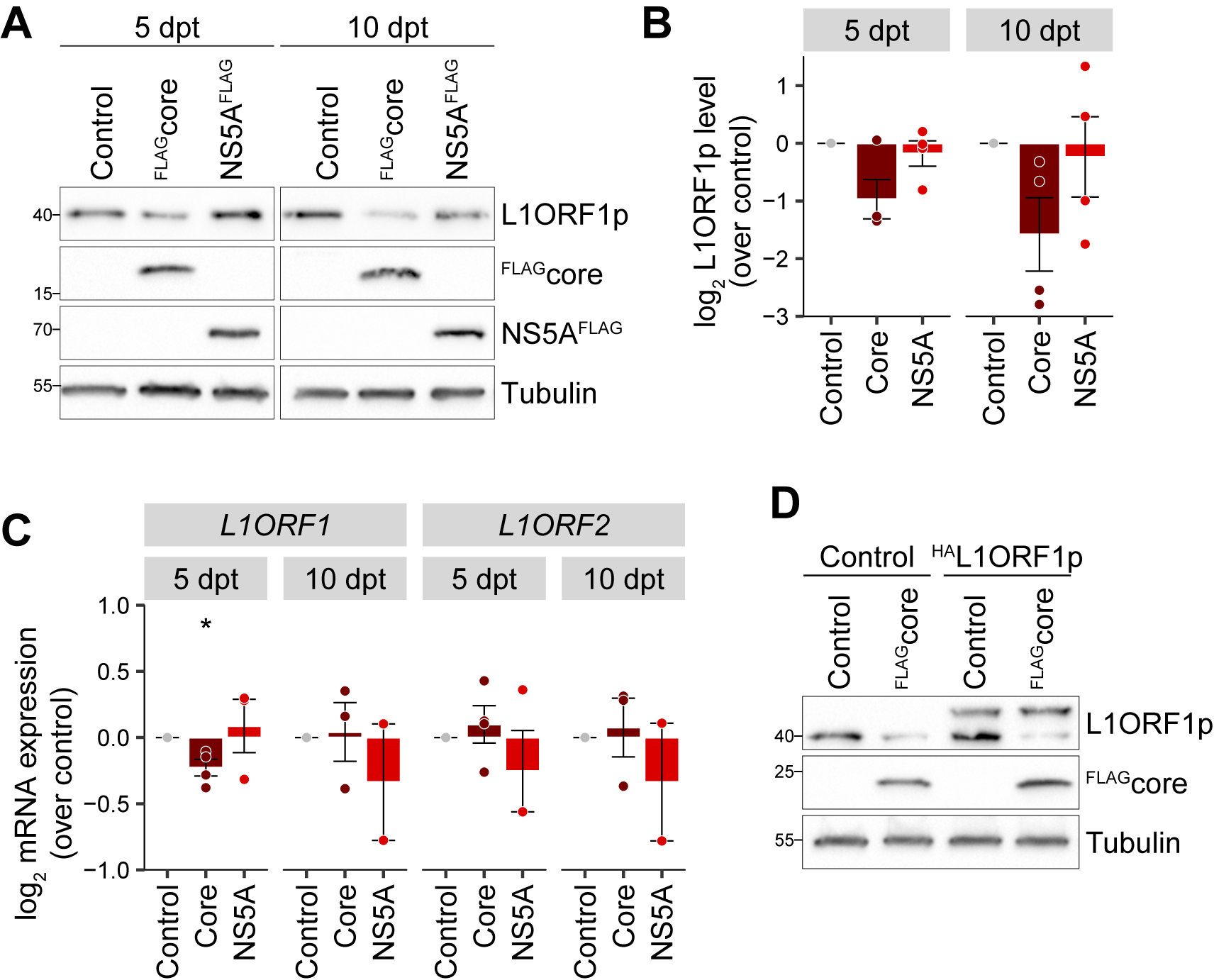

Supplement: S11 Fig — (A) HCV core expression decreases endogenous L1ORFp levels. FLAGcore, NS5AFLAG, or control lentivirus-transduced Huh7.5 cells were lysed at 5 and 10 dpt and endogenous L1ORF1p levels were analyzed by immunoblotting. Tubulin served as loading control. (B) Quantification of protein bands detected in (A) depicts the relative L1ORF1p levels normalized to tubulin (mean ± SEM, n = 4). (C) L1 mRNA levels were determined by qRT-PCR of FLAGcore, NS5AFLAG, or control lentivirus-transduced cells (mean ± SEM, nFLAGcore5 dpt = 4, nFLAGcore10 dpt = 3, nNS5AFLAG5 dpt = 3, nNS5AFLAG10 dpt = 2, *p< 0.05, Welch’s t-test). (D) Immunoblot analysis of Huh7.5 cells transduced with lentiviral constructs for the expression of HAL1ORF1p and FLAGcore. Tubulin served as loading control. Shown is one representative experiment (n = 3). (TIF) [file ppat.1009496.s011.tif]

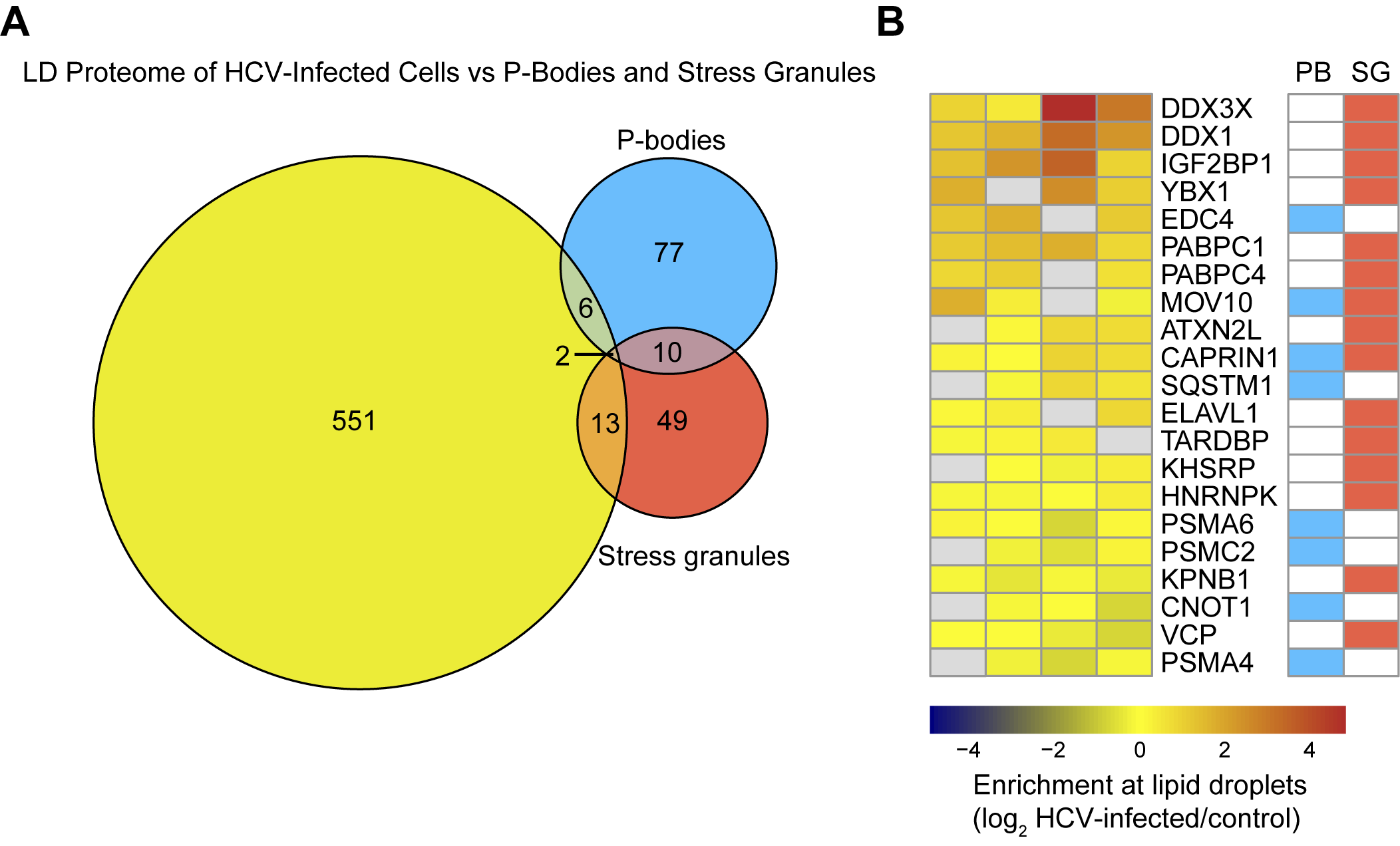

Supplement: S12 Fig — (A) Euler diagram of the overlap between the lipid droplet proteome dataset of HCV-infected cells from Rösch et al. [53] and annotated stress granule and P-body proteins (Downloaded from g:Profiler) [93]. (B) Heatmap depicting all proteins in the overlap with the lipid droplet proteome ordered according to enrichment in the lipid droplet fraction of HCV-infected vs. uninfected cells. Red, blue and gray coloring indicates enrichment, depletion, and ‘not applicable’, respectively. Right panel denotes if proteins are classified as P-body or stress granule protein. PB, P-body; SG, stress granule. (TIF) [file ppat.1009496.s012.tif]

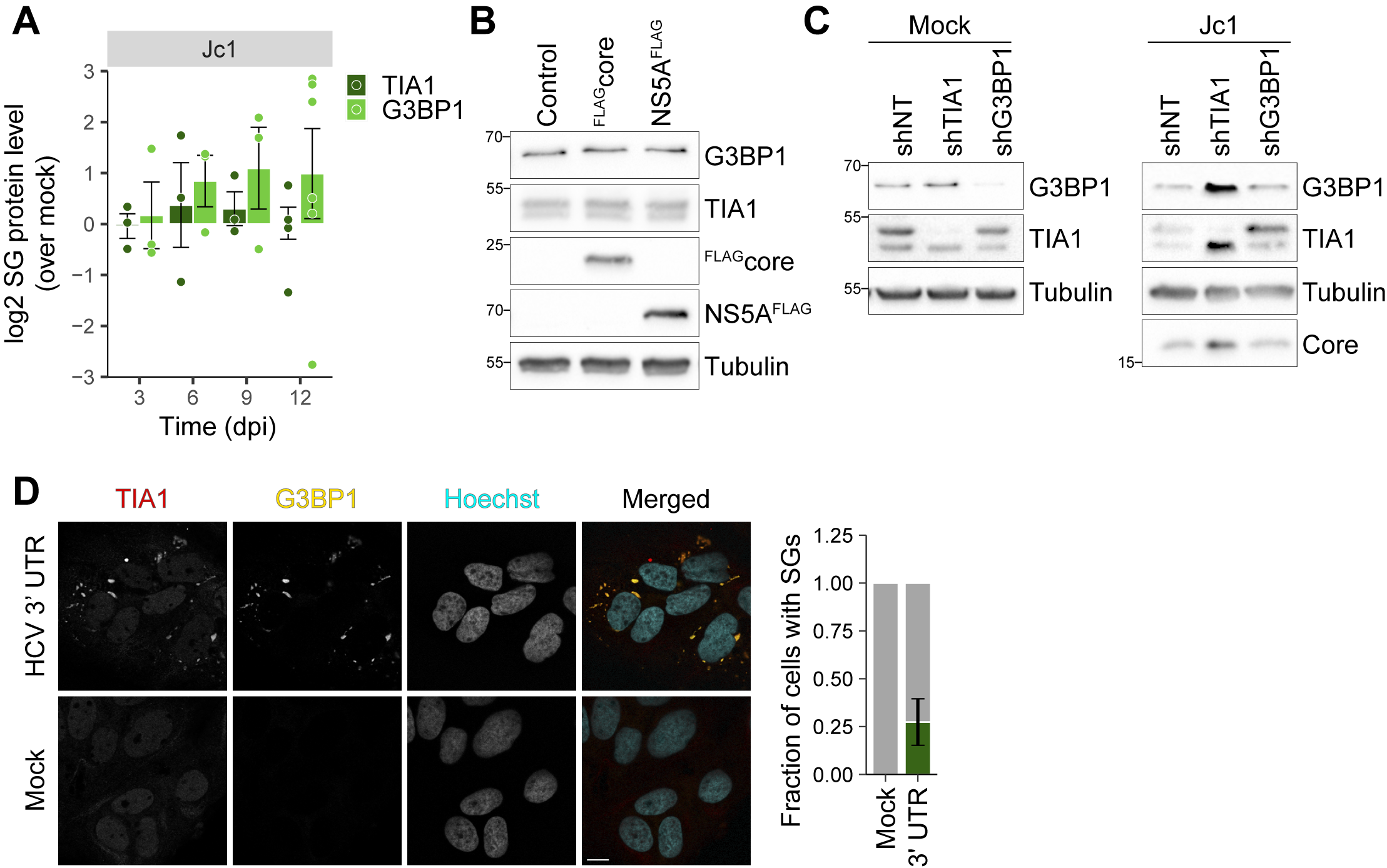

Supplement: S13 Fig — (A) Time course of TIA1 and G3BP1 protein levels during HCV infection. Huh7.5 cells were infected with Jc1NS5AB-EGFP (MOI = 0.2) or mock-infected, cells were lysed at the indicated time points and analyzed by immunoblotting using TIA1- and G3BP1-specific antibodies. HCV core expression was analyzed to confirm successful infection and tubulin served as loading control, respectively. For TIA1, the prominent upper band was quantified. Bar graph shows protein levels normalized to tubulin as fold over mock (mean ± SEM, n = 3–6). (B) Expression of HCV core and NS5A does not increase stress granule marker levels. Huh7.5 cells were transduced with lentiviral expression constructs for FLAGcore or NS5AFLAG and stress granule protein levels were analyzed by immunoblotting using TIA1- and G3BP1-specific antibodies. Tubulin served as loading control. Shown is one representative experiment (n = 5). (C) HCV infection counteracts shRNA-mediated TIA1 and G3BP1 knockdowns. Huh7.5 cells were transduced with lentiviral shRNA constructs targeting TIA1, G3BP1, or a non-targeting control (shNT). Cells were infected with Jc1NS5AB-EGFP or mock-infected and analyzed by immunoblot 13 dpi using TIA1- and G3BP1-specific antibodies. HCV core expression was analyzed to confirm successful infection and tubulin served as loading control (n = 3). (D) HCV 3‘ UTR RNA triggers stress granule formation. Huh7.5 cells were transfected with in vitro transcribed HCV JFH1 3‘ UTR RNA or mock-transfected and stained with G3BP1- and TIA1-specific antibodies and Hoechst. Shown are representative images (scale bar 10 μm). For quantification, fields were randomly selected and cells with TIA1/G3BP1-positive granules were counted as SG positive (# of cells from 2 independent experiments: n3’ UTR = 96; nMock = 103). (TIF) [file ppat.1009496.s013.tif]
